# Integrating experimental model, LC-MS/MS chemical analysis, and systems biology approach to investigate the possible antidiabetic effect and mechanisms of *Matricaria aurea* (Golden Chamomile) in type 2 diabetes mellitus

**DOI:** 10.3389/fphar.2022.924478

**Published:** 2022-09-07

**Authors:** Yassin Ismail, Dina M. Fahmy, Maivel H. Ghattas, Mai M. Ahmed, Walaa Zehry, Samy M. Saleh, Dina M. Abo-elmatty

**Affiliations:** ^1^ Department of Biochemistry, Faculty of Pharmacy, Suez Canal University, Ismailia, Egypt; ^2^ Natural Products Unit, Department of Medicinal and Aromatic Plants, Desert Research Center, Cairo, Egypt; ^3^ Department of Medical Biochemistry, Faculty of Medicine, Port Said University, Port Said, Egypt; ^4^ Department of Biochemistry, Faculty of Pharmacy, Mansoura University, Mansoura, Egypt

**Keywords:** network pharmacology, LC-MS/MS, Matricaria aurea (MA), diabetes, metabolic pathways, oxidative stress

## Abstract

Type 2 diabetes mellitus (T2DM) is a heterogeneous disease with numerous abnormal targets and pathways involved in insulin resistance, low-grade inflammation, oxidative stress, beta cell dysfunction, and epigenetic factors. Botanical drugs provide a large chemical space that can modify various targets simultaneously. *Matricaria aurea* (MA, golden chamomile) is a widely used herb in Middle Eastern communities for many ailments, including diabetes mellitus, without any scientific basis to support this tradition. For the first time, this study aimed to investigate the possible antidiabetic activity of MA in a type 2 diabetic rat model, identify chemical constituents by LC-MS/MS, and then elucidate the molecular mechanism(s) using enzyme activity assays, q-RTPCR gene expression analysis, network pharmacology analysis, and molecular docking simulation. Our results demonstrated that only the polar hydroethanolic extract of MA had remarkable antidiabetic activity. Furthermore, it improved dyslipidemia, insulin resistance status, ALT, and AST levels. LC-MS/MS analysis of MA hydroethanolic extract identified 62 compounds, including the popular chamomile flavonoids apigenin and luteolin, other flavonoids and their glycosides, coumarin derivatives, and phenolic acids. Based on pharmacokinetic screening and literature, 46 compounds were chosen for subsequent network analysis, which linked to 364 candidate T2DM targets from various databases and literature. The network analysis identified 123 hub proteins, including insulin signaling and metabolic proteins: IRS1, IRS2, PIK3R1, AKT1, AKT2, MAPK1, MAPK3, and PCK1, inflammatory proteins: TNF and IL1B, antioxidant enzymes: CAT and SOD, and others. Subsequent filtering identified 40 crucial core targets (major hubs) of MA in T2DM treatment. Functional enrichment analyses of the candidate targets revealed that MA targets were mainly involved in the inflammatory module, energy-sensing/endocrine/metabolic module, and oxidative stress module. q-RTPCR gene expression analysis showed that MA hydroethanolic extract was able to significantly upregulate PIK3R1 and downregulate IL1B, PCK1, and MIR29A. Moreover, the activity of the antioxidant hub enzymes was substantially increased. Molecular docking scores were also consistent with the networks’ predictions. Based on experimental and computational analysis, this study revealed for the first time that MA exerted antidiabetic action *via* simultaneous modulation of multiple targets and pathways, including inflammatory pathways, energy-sensing/endocrine/metabolic pathways, and oxidative stress pathways.

## Introduction

Type 2 Diabetes Mellitus T2DM is a multifarious metabolic syndrome that has a strong association with hyperglycemia, hyperlipidemia, and obesity. It encompasses a lot of disturbances, including defective metabolism of carbohydrates, lipids, and proteins; oxidative stress; chronic low-grade inflammation; and epigenetic factors, such as regulatory microRNAs. T2DM has two major pathophysiological arms: resistance to the action of insulin, primarily in the liver and other insulin-sensitive tissues; and pancreatic beta cell secretory dysfunction (Czech, 2017; Galicia-Garcia et al., 2020). Abnormal Changes in signaling proteins associated with insulin signaling clearly play a crucial part in the insulin resistance state. Diabetes patients have insulin signal transduction pathway changes or defects, which are associated with lower levels of the molecules of insulin signaling such as “Insulin receptor substrate-1 (IRS-1) and phosphatidylinositol 3-kinase PI3K” (Dhanya et al., 2017). Hepatic insulin resistance causes increased glucose production from the liver owning to the failure of insulin signal to modulate the transcription factor foxo1, and thus fails to suppress transcription of phosphoenolpyruvate carboxy kinase (PEPCK or PCK1) and the gluconeogenesis pathway (Peng et al., 2020; Ge et al., 2021). Upregulation of PEPCK, the rate limiting enzyme of gluconeogenesis, takes place in almost all diabetes models as a result of a lack of insulin and/or its action. A large body of evidence suggests a direct correlation between PEPCK expression, insulin resistance, and glycemic status (Elo et al., 2007; Gómez-Valadés et al., 2008; Xu et al., 2019).

Oxidative stress linked to obesity and overnutrition has been proposed as a single unifying mechanism underlying insulin resistance, beta cell dysfunction, glucose intolerance, development of T2DM, and progression of the disease leading to macro and micro complications (Ceriello and Motz, 2004; Wright et al., 2006; Tangvarasittichai, 2015; Burgos-Morón et al., 2019). Oxidative stress is a covariate of glucose in both the development of diabetes and recovery during antidiabetic therapy. In addition, oxidative stress and antioxidant biomarkers show a strong correlation with glycemic status and glucose metabolism (Kulkarni et al., 2014). Moreover, oxidative stress increases PEPCK expression in the liver, which is a hallmark of hepatic insulin resistance (Ito et al., 2006). Overnutrition and its consequences, such as oxidative stress, potentiate the production of inflammatory cytokines, particularly IL-1β, TNF-α, IL-6, and many other cytokines and chemokines that are IL-1β-dependent. The activation of nuclear factor kappa B “NF-κB” in the liver, which controls the generation of proinflammatory cytokines like IL-1β, is thought to cause local and systemic insulin resistance (Cai et al., 2005; Rehman and Akash, 2016; Oguntibeju, 2019).

Another player in the arena of T2DM biology is microRNAs, which have emerged as etiological agents, diagnostic and prognostic biomarkers in T2DM, and promising therapeutic targets. MicroRNA 29a has been reported to be one of the strongest candidate Mirna regulatory hubs in the T2DM gene network, as well as one of the most upregulated miRNAs in various insulin-sensitive tissues in T2DM patients and rodent models of diabetes. Several studies have found that abnormally high expression of hepatic miRNA 29a in high-fat diet and genetic animal models is causally linked to insulin resistance, hyperlipidemia, and prevents insulin from downregulating PEPCK, resulting in unrestrained gluconeogenesis. Molecularly, Mirna 29a targets the regulatory unit p85 of PI3-K, a key signaling molecule in the insulin transduction pathway, causing insulin signal truncation, and contributing to insulin resistance (He et al., 2007; Pandey et al., 2011; Baran-Gale et al., 2013; Kurtz et al., 2014, 2015; Bhatia et al., 2015; Zhu and Leung, 2015; Dooley et al., 2016).

Given the increased incidence of T2DM (Khan et al., 2020) and the failure of current drugs to control the multiple pathophysiological processes in the long term, it is necessary to move forward in the search for novel medications that provide better solutions for the multiple abnormal targets and pathways of this disease (Gomez-Peralta and Abreu Padín, 2014). Plants reported in traditional medicine to possess antidiabetic activity provide an interesting and wealthy resource to find new drugs in their hugely diverse compounds. Historically, the development of the majority of established drugs, including the blockbuster drug metformin, was based on the identification of compounds from natural sources (Witters, 2001; Li and Vederas, 2009). Since ancient times, the Egyptians, Greeks, Romans, and Arabs have used chamomile, one of the most popular herbs in the world. *Matricaria aurea*, also known as golden chamomile (Soubra et al., 2018), is reported to be used in traditional medicine in some Middle Eastern countries as an antidiabetic herb (Yaniv et al., 1987; Al-Mustafa and Al-Thuniba, 2008). It is also widely used as a therapy for a variety of other conditions, including inflammation, respiratory disorders, facilitating delivery during pregnancy, and infant health care (Ali-Shtayeh et al., 2015; Abdelhalim et al., 2017). Previous experimental work, which is mainly *in vitro*, reported the antioxidant (Mohammad Al-Ismail and Aburjai, 2004), anti-inflammatory (Khodadadi et al., 2011), anti-ulcerative colitis (Minaiyan et al., 2011), analgesic (Qnais, 2011), antibacterial, and anticancer (Kheder et al., 2014; Ahmad et al., 2020, 2021) biological activity of *Matricaria aurea*. To the best of our knowledge, there is no scientific study that has investigated the antidiabetic activity of this plant.

Botanical extracts with numerous constituents may elicit pharmacological activity by modulating a variety of targets and signaling pathways, which could help in the treatment of T2DM. However, the complexities of herbal extracts may render the mission to discover the underlying molecular mechanisms more difficult. In order to overcome this problem, a new research methodology called network pharmacology has been developed to find the targets of drugs and elucidate mechanism(s) of action. Network pharmacology is a new theoretical framework and methodology grounded on the advancement of omics technologies, bioinformatics, systems biology, mathematics, and pharmacology to examine the impact of drugs on both the interactome and diseasome levels. Instead of the old paradigm of “one drug-one target-one disease,” network pharmacology adopts a new paradigm of “one or multi drugs-multi targets or multiple-component-therapeutics/network-targets.” This approach makes it extremely useful for analyzing drug combinations, especially botanical formulas (Chandran et al., 2017; Li et al., 2017; Zhang et al., 2019).

**FIGURE 1:**
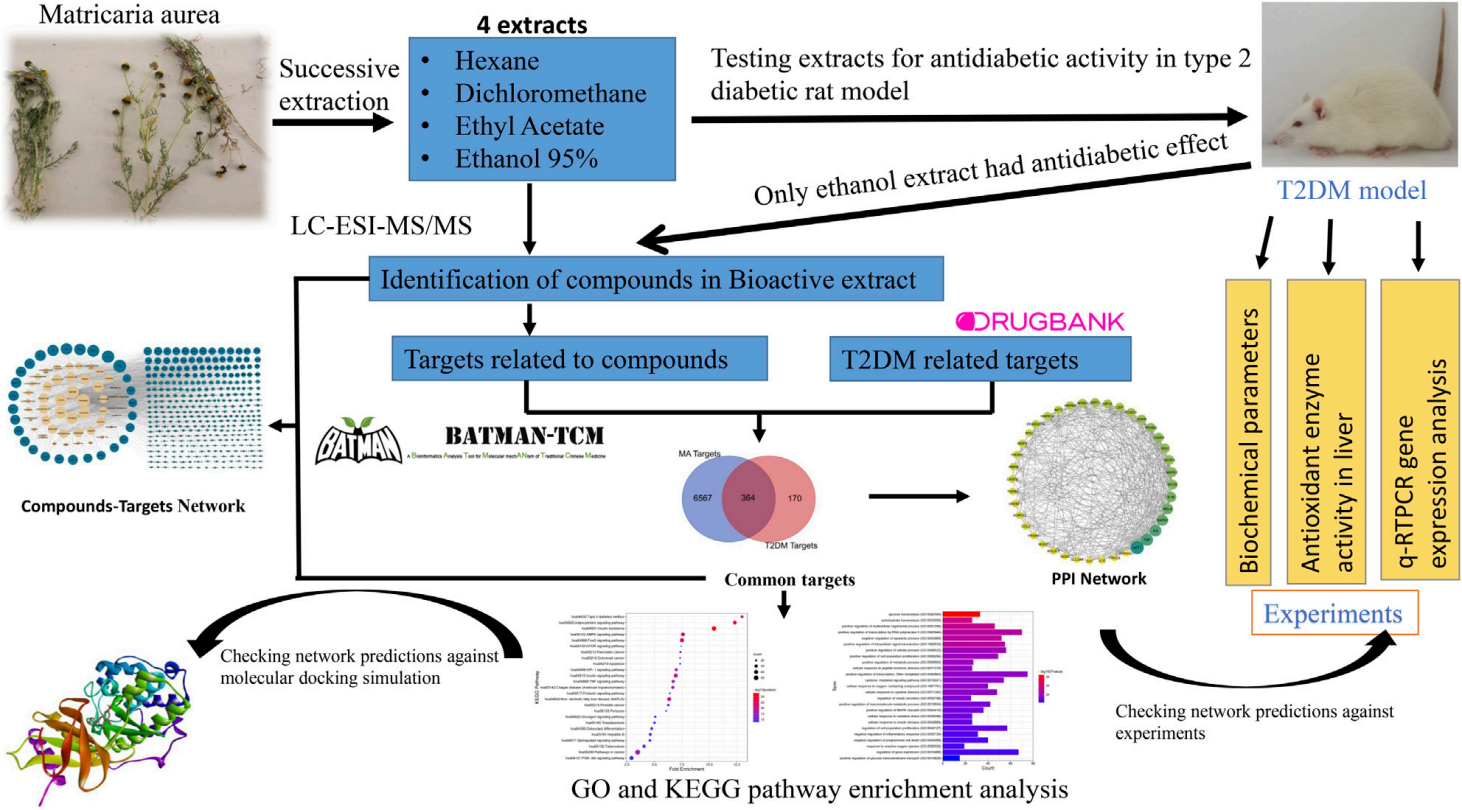
Graphical abstract. Adjusting the size of the images, resolution, and combining the images was carried out using Photoshop.

In this article, we report for the first time the antidiabetic activity of the polar hydroethanolic extract of *Matricaria aurea.* Furthermore, it improved dyslipidemia, oxidative stress status, and gene expression of important genes related to T2DM in an experimental rat model, and the active constituents were identified using the UPLC-Q-TOF MS/MS system. Moreover, we used network pharmacology principles and methods to decipher the potential mechanism of action of MA in T2DM treatment. Finally, we checked the reliability of network predictions against molecular docking simulation, q-RTPCR data, and enzyme activity assay data, as shown in Figure 1. Our findings lay the theoretical basis for the development of new antidiabetic drugs, more detailed mechanistic studies, and clinical trials.

## Materials and methods

### Plant material

The aerial parts of *Matricaria aurea* (Loefl.) Sch. Bip. (Family: Asteraceae) were collected from its wild habitat in Wadi Habis (Latitude 31° 22′ 81″N and Longitude 27° 3′ 54″E), Mersa Matruh governorate, Northwestern Coast, Egypt. The plant identity was identified and authenticated by Dr. Omran Ghaly, Head of the Plant Taxonomy Unit, Desert Research Center, Matria, Cairo, Egypt. A voucher specimen was deposited at the herbarium of the Desert Research Center (CAIH) with code number: CAIH-970-R.

#### Preparation of *Matricaria aurea* extracts

MA aerial parts were air-dried in the shade then crushed into a powder (3.45 kg). The MA powder was successively extracted as described by (Pérez-Bonilla et al., 2013). In a Soxhlet apparatus (500 ml), four different solvents were used in ascending order of polarity (n-hexane, dichloromethane, ethyl acetate, and ethanol (95%)). All extracts of various organic solvents were dried by evaporating each solvent at 40°C using a rotary evaporator (R-205-Buchi, Germany), yielding 102.4 g of ethanol 95% extract, 24 g of ethyl acetate extract, 27.9 g of dichloromethane extract, and 45.2 g of hexane extract, respectively. In this study, two doses of 100 and 200 mg/kg were selected and used (Minaiyan et al., 2011; Kaseb et al., 2018; Hajizadeh-Sharafabad et al., 2020). The two doses were prepared by dissolving an appropriate amount of the dried extracts in 1 ml of Tween 20. Following that, 9 ml of 0.9% NaCl was added to each mixture. To prepare the vehicle, 1 ml of Tween 20 was dissolved in 9 ml of 0.9% NaCl.

### Experimental animals

Healthy male Wistar rats, 5 weeks old and weighing 90–110 g were obtained from the national research center (Cairo, Egypt). Rats were left for 1 week for acclimatization before beginning the experiment. Throughout the experiment, rats were maintained on 12 h light/dark cycle and temperature control (20–24°C). The animals were fed a standard chow diet and given water on an *ad libitum* basis throughout the experimental period, except when otherwise stated. Animal handling and experimental procedures were approved by the Research Ethics Committee of the Faculty of Pharmacy, Suez Canal University, Ismailia, Egypt (Code: 201803RA1), which are in accordance with the Guide for the Care and Use of Laboratory Animals (National Research Council, 2011).

#### Experimental induction of type 2 diabetes using high fat diet and low dose of streptozotocin “STZ”

Rats were divided into two dietary regimens at the beginning of the experiment: normal pellet diet (NPD; 67% carbohydrate, 21% protein, and 12% calories) and high fat diet (HFD; 17% carbohydrate, 25% protein, and 58% fat, as a percentage of total kcal) (detailed composition and energy density for both diets are presented in Supplementary Data Sheet S1) (Abo-elmatty et al., 2013). After 4 weeks of diet manipulation, rats fed on the HFD, were given a single intraperitoneal injection of 40 mg/kg/body weight of streptozotocin (STZ; Cat No: SLBJ7785V, Sigma Aldrich, CHEMIE) dissolved in di-sodium citrate buffer (pH 4.5), while normal non-diabetic rats received a single intraperitoneal injection of the vehicle (Skovsø, 2014). Ten days following STZ injection, fasting glucose levels were estimated in blood samples from the tail tip using the glucometer method (Accutrend alpha-Roche Diagnostic, Germany). Only rats with fasting blood glucose level >200 mg/dl were considered diabetic and included in the study.

#### Experimental design

Seventy diabetic rats were randomly assigned into 10 groups of seven animals each. Nine groups received either pioglitazone (standard drug, PIO) at a dose of 20 mg/kg (Abo-elmatty et al., 2013) or one of two doses at 100 and 200 mg/kg of MA extracts: 95% ethanol extract (ETH) or ethyl acetate extract (EA) or dichloromethane extract (DCM) or hexane extract (HEX). The remaining group served as the diabetic control group (DC). In addition, another group (seven rats) without induction of diabetes served as a normal control group (NC) and received the vehicle. Starting on the 11th day after diabetes was induced, the vehicle, the standard drug, and plant extracts were given once a day by oral gavage for 4 weeks.

#### Blood sampling and biochemical analysis

Rats were fasted overnight, anesthetized with diethyl ether, and euthanized at the end of the experiment. Retroorbital blood samples were taken, and liver samples were collected and kept at −80°C for RNA extraction and enzyme activity assays. To obtain serum samples, blood samples were subjected to centrifugation at 3000 rpm for 15 min, and then the serum was stored at −80°C until the biochemical assay.

##### Assessment of fasting blood glucose, serum insulin, insulin resistance and insulin sensitivity

Fasting blood glucose and serum insulin were estimated using the glucometer method (Accutrend alpha, Roche Diagnostic GMbH, Germany) and rat insulin ELISA kit (Wuhan Fine Biotech Co., Ltd., China), respectively. Systemic insulin resistance was estimated using the homeostasis model assessment index for insulin resistance (HOMA-IR) using the following equation: HOMA−IR index=[fasting glucose (mg/dl)×fasting insulin (uU/ml)] / 405) . The triglyceride and glucose (TyG) index, is an important predictor for fatty liver and hepatic insulin resistance. This index was estimated using the following formula TyG index=Ln [ triglycerides(mg/dl)×fasting glucose (mg/dl)/ 2] . To determine insulin sensitivity, the quantitative insulin sensitivity check index (QUICKI)=1/ [log⁡ fasting insulin (uU/ml)+log⁡ fasting glucose (mg/dl)] was used. To determine the secretory capacity of the pancreas, HOMA-B index was calculated as follows: HOMA−B index=20×fasting insulin (uU/ml)/ [fasting glucose (mg/dl)−63] (Abo-elmatty et al., 2013; Yoon et al., 2016; Zhang et al., 2017).

##### Assessment of lipid profile and liver injury markers

Serum total cholesterol (TC), triglycerides (TGs), high density lipoprotein-cholesterol (HDL-C), low density lipoprotein-cholesterol (LDL-C), alanine aminotransferase (ALT), and aspartate aminotransferase (AST) were calorimetrically quantified using commercial kits from (Biodiagnostic, Giza, Egypt) according to the spectrophotometric methods described in the manufacturer’s protocol.

##### Oxidative stress and antioxidant markers in liver

To determine oxidative stress and antioxidant markers in the liver tissue, malondialdehyde (MDA) (Ohkawa et al., 1979), reduced glutathione (GSH) (Owens and Belcher, 1965) levels as well as catalase (Fossati et al., 1980) and superoxide dismutase (SOD) (Nishikimi et al., 1972) activities were measured using commercial kits from (Biodiagnostic, Giza, Egypt) according to the manufacturer’s protocol.

##### qRT-PCR analysis

Isolation of total RNA, including miRNAs, from liver tissue was carried out using the Qiagen miRNeasy Mini kit (Cat No. 217004; Qiagen, Hilden, Germany) according to the supplied manufacturer’s protocol. RNA concentration and purity were measured spectrophotometrically using the Nano Drop ND-1000 spectrophotometer (NanoDrop Tech., Inc., Wilmington, DE, United States). RNA was reverse transcribed to cDNA by the High-Capacity cDNA Reverse Transcription Kit, which uses the MultiScribe^™^ Reverse Transcriptase (Cat. No. 4368814, Applied Biosystems, Egypt). RT was carried out in a Mastercycler Gradient Thermocycler (Eppendorf, Hamburg, Germany) using the following parameter values: 25°C for 10 min, followed by 37°C for 120 min, and finally, 85°C for 5 min, then held at 4°C. While mirna-29a was reverse transcribed using the TaqMan^™^ MicroRNA Reverse Transcription kit and a miRNA-29a specific stem–loop primer (Applied Biosystem, assay ID 002112, Egypt). The Thermocycler was programmed according to the following conditions: 16°C for 30 min, 42°C for 30 min, and 85°C for 5 min, then held at 4°C to perform miRNA RT. The relative expression of PCK1, p85α PIK3R1, IL-1β and miRNA-29a was quantified using the TaqMan primers and probes with the following assay IDs (Applied Biosystems, assay IDs Rn01529014_m1 for PCK1, Rn01644964_m1 for Pik3r1, Rn00580432_m1 for IL-1β and 002112 for miRNA-29a). 18s rRNA was used as an endogenous control (Applied Biosystems, assay ID Hs99999901_s1). The PCR was performed on the StepOne^™^ Real-Time PCR System (Applied Biosystems) with the following conditions: 50°C for 2 min, then 95°C for 10 min, followed by 40 cycles of 95°C for 15 s and 60°C for 1 min. Relative gene expression was calculated by the 2^−ΔΔCt^ method after normalization to 18s rRNA (Toraih et al., 2015, 2019; Abd Allah et al., 2021).

### UPLC-QTOF-MS/MS analysis of hydroethanolic extract

#### Sample preparation

A stock solution of the extract was made by dissolving 50 mg of aq.-ethanolic extract (5:95) in 1 ml of a (2:1:1) solvent mixture of water, methanol, and acetonitrile (H_2_O: MeOH: ACN). To obtain complete solubility of the stock solution, the sample was vortexed and ultra-sonicated at 30 kHz for 10 min. 20 µl aliquot of the stock solution was again diluted with 1000 µl of the (2:1:1) H_2_O: MeOH: ACN, centrifuged at 10,000 rpm for 5 min before injection, and 10 µl (1 μg/ml) was used. For experiment confidence, the LC-MS analysis was also performed on blank and quality control samples/internal standard (IS). In both positive and negative modes, the sample was injected.

#### Instrument and data acquisition

UPLC separation of phytochemicals was carried out on a reversed-phase Exion Xbridge C18 column (2.1 50 mm, 3.5 m) from (Waters Corporation, Milford, MA, United States), which was preceded by an in-line filter disks pre-column (0.5 µm × 3.0 mm, Phenomenex, Torrance, CA, United States). For each mode, the mobile phase contained two solvents: solvent (A) 5 mM ammonium formate in 1% methanol, pH adjusted to 3.0 by using formic acid, solvent (B) 5 mM ammonium formate in 1% methanol, adjusted to pH 8 using sodium hydroxide, and solvent (C) 100% acetonitrile. For the positive ion mode, solvents (A) and (C) were used, while for the negative ion mode, solvents (B) and (C) were used. The column temperature was maintained at 40°C, and the flow rate was set to 300 μl/min. The gradient elution was performed with the following parameters: 0–20 min, (90% (A) or (B), 10% (C); 21–25 min, (10% (A) or (B), 90% (C); 25.01–28 min, (90% (A) or (B), 10% (C)). (A) was used for the positive ion mode only, while solvent (B) was used for the negative ion mode only.

The mass spectrometry (MS) was carried out on a Triple TOF 5600+ system with a Duo-Spray source in the ESI mode (AB SCIEX, Concord, ON, Canada). In the positive mode, the sprayer capillary and declustering potential voltages were 4500 and 80 eV, respectively, and in the negative mode, -4500 and −80 V. The collision energies of 35 V (positive mode) and −35 V (negative mode) were used, together with CE spreading of 20 V and ion tolerance of 10 ppm. The TripleTOF5600+ was operated using an information-dependent acquisition (IDA) protocol. Analyst-TF 1.7.1 was used to collect batches of MS and MS/MS data. The IDA method was utilized to simultaneously collect full-scan MS and MS/MS information. The method used high-resolution survey spectra ranging from 50 to 1100 m/z, and the mass spectrometer was programmed to detect a 50-ms survey scan. Following that, the top 15 most intense ions were chosen for MS/MS fragmentation spectra acquisition after each scan (El Sayed et al., 2020; Mohammed et al., 2021).

#### LC-MS data processing

For non-targeting, small molecule comprehensive analysis of the sample, MS-DIAL 3.70 open-source software was utilized. ReSpect positive (2737 records) or ReSpect negative (1573 records) databases were used as reference databases, depending on the acquisition mode. The search parameters were set as MS1 and MS2 mass tolerance: 0.01 and 0.05 Da for data collection, for peak detection; minimum peak height: 100 amplitude, mass slice width: 0.05 Da, smoothing level: two scans, minimum peak width: six scans, for identification MS1 and MS2 tolerance: 0.2Da/each, for alignment; retention time tolerance: 0.05 min and MS1 tolerance: 0.25 Da (El Sayed et al., 2020; Mohammed et al., 2021).

### Data preparation for network pharmacology analysis

#### Screening of MA bioactive compounds based on physicochemical and pharmacokinetic properties

Compounds identified based on UPLC/MS-MS were further screened for oral bioavailability and drug likeness. Using accurate searching canonical SMILES, important pharmacology-related properties were obtained from the Swiss ADME tool (http://www.swissadme.ch/), including molecular weight, Log Po/w, hydrogen bond acceptor count, hydrogen bond donor count, and number of rotatable bonds. Based on the rule of five, these properties were applied to the drug likeness evaluation. The candidate bioactive chemicals were selected if they complied with two or more of the five drug-likeness filters (Lipinski, Veber, Ghose, Muegge, Egan) and had a bioavailability score of 0.55 or more, as proposed by the Swissadme tool, which allows for the computation of physicochemical properties and prediction of druglike nature, pharmacokinetic properties, and ADME parameters (Daina et al., 2017).

#### Collection of targets related to MA active constituents

Six databases containing information about drug or chemical interactions with biological targets were used in this study to retrieve targets of MA active constituents. These databases include: Binding Database (https://www.bindingdb.org/bind/chemsearch/marvin/FMCT.jsp) retrieving targets matched only to “exact” compound and affinity filter of no more than 10,000 nM, Swiss Target Prediction (http://www.swisstargetprediction.ch/) with a score cutoff = 0.5, STITCH database (http://stitch.embl.de/) with a score cutoff 0.4, Therapeutic Target database (TTD, http://db.idrblab.net/ttd/), Comparative Toxicogenomics database (CTD, http://ctdbase.org/), and the first bioinformatics analysis tool for molecular mechanism of traditional Chinese medicine BATMAN-TCM (http://bionet.ncpsb.org.cn/batman-tcm/) with a target prediction score cutoff 20 (details of this work are presented in Supplementary Table S4).

#### Collection of known targets related to T2DM disease

To retrieve the known targets associated with T2DM, we used the key words “Type 2 Diabetes Mellitus” and “Insulin Resistance” to search in the following four databases: Online Mendelian Inheritance in Man (OMIM, https://www.omim.org/), DisGeNET (version 7.0) with a score cutoff of 0.2 (https://www.disgenet.org/), Therapeutic Target Database (TTD, http://db.idrblab.net/ttd/), and DRUGBANK (only targets for FDA approved drugs) (http://www.drugbank.ca). Targets were converted into HUGO Gene Nomenclature Committee (HGNC) approved gene symbols and then merged (details of this work are presented in Supplementary Table S4).

#### Protein-protein interaction of the common targets between T2DM targets and MA targets

To get overlapping targets between T2DM disease targets and MA chemical constituents’ targets, we used the online tool “calculate and draw custom Venn diagrams” (https://bioinformatics.psb.ugent.be/cgi-bin/liste/Venn/calculate_venn.htpl) to create a Venn diagram and analyze the intersection between the two sets of targets. The intersection between MA targets and T2DM targets was subsequently utilized to build our compound-target network and protein-protein interaction network and was considered as MA candidate targets against T2DM. To get protein-protein interaction data, overlapping targets were further used in the STRING database (version 11.5, https://string-db.org/), and the screening parameters used were “Homo sapiens,” with a high interaction score of 0.7 as the minimum interaction score.

### Networks construction, visualization and analysis

To decode the mechanism of action of MA against T2DM, we constructed the following three main networks: *1*) The Compounds-targets (C-T) network, which links MA compounds and their overlapping targets with T2DM. *2*) The Target-Target or Protein-Protein interaction (T-T) network of common targets for both MA and T2DM common targets. *3*) Compound-Core key target-Pathway (C-T-P) network.

All the networks were constructed and visualized using Cytoscape (version 3.9.1), which is an open-source software for visualizing interactions between molecules, biomolecules, and biological pathways. In addition, many apps can work efficiently with cystoscope to analyze the network based on the equations of graph theory. Topological analysis was done using the network analyzer plug-in in Cytoscape. The fundamental units of a network are nodes and edges. Nodes represent drugs, proteins, or pathways, and edges represent interactions between them. Three topological properties, including degree centrality, closeness centrality, and betweenness centrality, were used to judge the importance of targets and compounds in the networks. The standard definition of degree is the number of direct neighbors of a node. The greater the number of a node’s direct links or edges, the greater its degree and influence in the network. A node’s betweenness centrality is defined as the number of shortest paths between all pairs of nodes that pass through the node. It represents the extent to which nodes stand between each other and is often used to find nodes that serve as a bridge from one part of a graph to another. Closeness centrality is considered a measure of how long it will take to spread information sequentially from one node to all other nodes. The higher the degree/closeness/betweenness centrality of a protein, the greater its importance in the protein-protein interaction network (Zhang et al., 2020; Que et al., 2021).

### Gene ontology and pathway enrichment analysis

Enrichment of Gene ontology (GO) terms; biological process (BP), molecular function (MF), and cellular component (CC) to understand genes’ functions was performed using Enricher (https://maayanlab.cloud/Enrichr). To understand the molecular mechanisms and pathways of our targets, Kyoto Encyclopedia of Genes and Genome (KEGG) pathway enrichment analysis was conducted on all 364 candidate targets by the David tool (https://david.ncifcrf.gov/home.jsp).

### Molecular docking

The ligands’ SDF files were retrieved from PubChem and converted to PDB format using open Babel software. The protein data bank (https://www.rcsb.org) was utilized to retrieve 11 PDB file formats of the core target proteins predicted by network analysis. The protein names and PDB IDs are as follows: AKT1 (1unq), AKT2 (1o6l), HIF1A (1h2m), IL6 (1alu), MAPK1 (6rq4), MAPK3 (4qtb), PPARA (2p54), PPARG (5y2o), SIRT1 (4zzi), TNF (2az5), and RELA (2ram). Ligands and proteins were further prepared for docking and converted to PDPQT file format using Biovia Discovery Studio and Autodock Tools. The Autodock Vina software was used to perform the docking simulation process, and the ligands with the lowest binding energy scores were chosen to visualize their interactions with proteins in the Biovia Discovery Studio software (Oh et al., 2020; Ren et al., 2021).

### Statistical analysis and graphing

Experimental data were presented as Mean ± SEM after analysis by the Statistical Package of Social Sciences (SPSS) program version 22, (Chicago, IL, United States). One-way analysis of variance (ANOVA) followed by a Tukey’s multiple comparison test was performed for comparisons among groups. For a specific value to be regarded a statistically significant, the *p* value must be less than 0.05. Graphs were created by GraphPad prism 9. Adjusting the size of the images and resolution, adding the panels’ letters, combining the images, and putting numbers on peaks were done using Adobe Photoshop CC (2019, V20.07.28362).

## Results

### Effect of MA on hyperglycemia, insulin resistance, hyperlipidemia and liver injury

**FIGURE 2:**
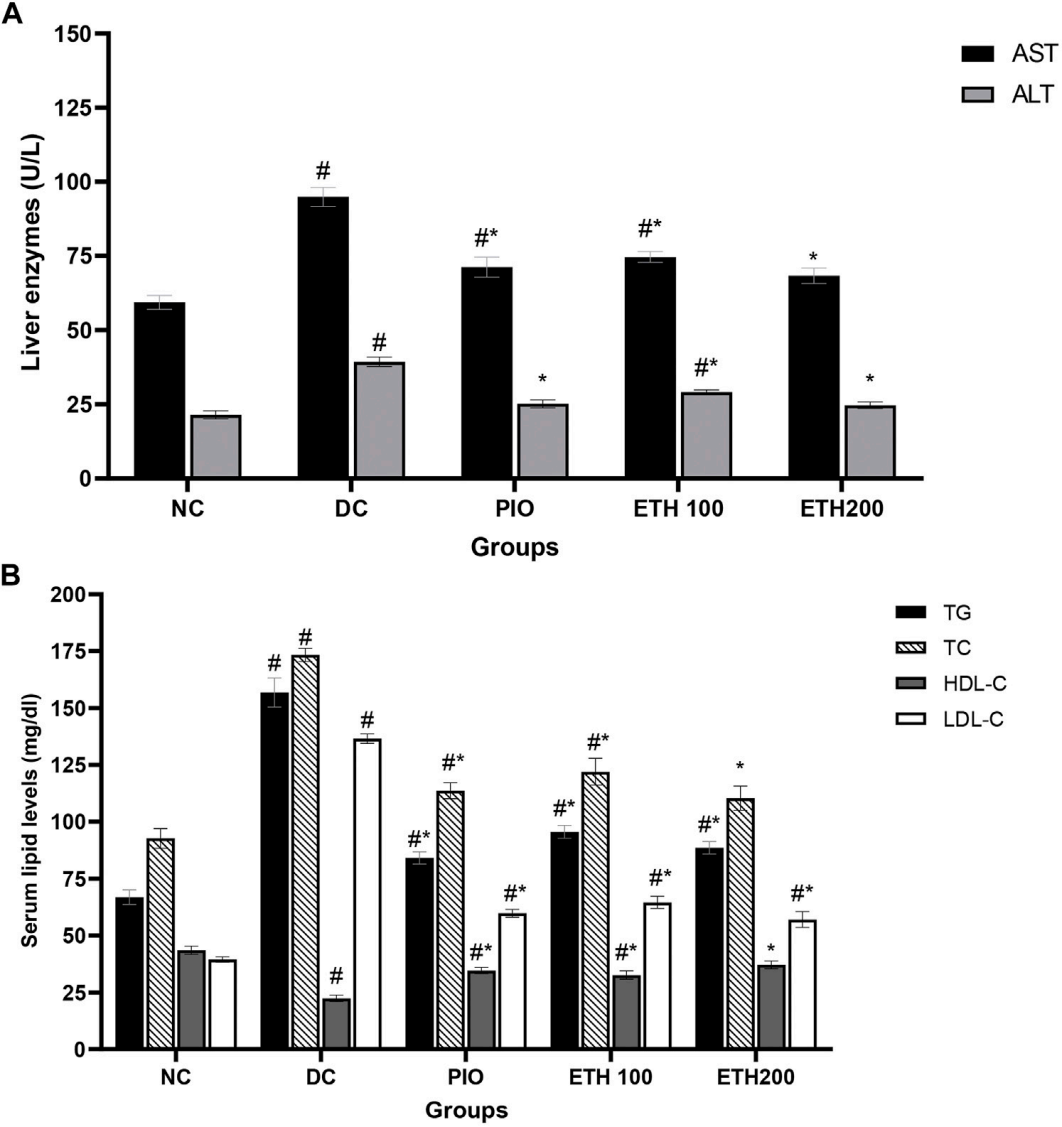
MA ethanolic extracts ameliorated the liver damage and dyslipidemia **(A)** liver enzymes: ALT, alanine aminotransferase; AST, aspartate aminotransferase, #*p* < 0.05 versus normal control, *p < 0.05 versus diabetic control. **(B)** Serum lipids: TG, triglycerides; TC, total cholesterol; LDL-C, low density lipoprotein cholesterol; HDL-C, high density lipoprotein cholesterol. #*p* < 0.05 versus normal control, *p < 0.05 versus diabetic control. Data are expressed as Mean ± SEM, *n* = 7. Adjusting the size of the images and resolution, adding the panels’ letters, and combining the images was carried out using Photoshop.

As shown in Table 1, rats on HFD + STZ showed a significant increase in blood glucose level compared to normal non-diabetic control group. The curative benefits of the four plant extracts were investigated in HFD + STZ-induced diabetic rats, and only MA hydroethanolic extract treatment at two different doses demonstrated a promising antihyperglycemic effect, as evidenced by a significant drop in fasting blood glucose levels. After 4 weeks of therapy, MA ethanolic extracts and pioglitazone treated rats had considerably lower glucose levels than the diabetic control. In contrast, the hexane, dichloromethane, and ethyl acetate extracts did not show significant improvement in blood glucose levels. This result strongly suggested that the antidiabetic activity of MA was concentrated in the ethanolic extract, which directed biochemical and phytochemical analysis to focus on it. The antihyperglycemic effect of pioglitazone did not show a statistical difference from MA ethanolic extracts. In addition, diabetic rats showed a significant reduction in fasting serum insulin, secretory capacity of the pancreas (HOMA-B index), and the index of insulin sensitivity QUICKI, while the index of insulin resistance HOMA-IR, triglyceride and glucose (TyG) index, and the markers of liver injury ALT and AST increased significantly in comparison to the normal control rats, as shown in Table 2 and Figure 2A, respectively. Moreover, as depicted in Figure 2B, serum lipids, including triglycerides, cholesterol, and LDL-C increased significantly, whereas HDL-C decreased in diabetic control rats compared to normal control rats. Treatment with either MA ethanolic extract or pioglitazone was successful in ameliorating insulin resistance, hyperlipidemia, and fatty liver without having a significant effect on fasting insulin levels or pancreatic secretory capacity. Furthermore, it increased liver protection from injury associated with T2DM, as evidenced by a significant decrease in ALT and AST.

**TABLE 1:** Effect of *Matricaria aurea* (MA) extracts on Fasting blood glucose level.

Groups	Fasting blood glucose (FBG) mg/dl
Normal Control (NC)	89.71 ± 4.21
Diabetic control (DC)	343.86 ± 10.65 ^#^
Diabetic + Pioglitazone (PIO)	179.86 ± 5.15 ^*^
Diabetic + Ethanol extract (ETH) 100 mg/kg	193.86 ± 4.66 ^*#^
Diabetic + Ethanol extract (ETH) 200 mg/kg	184.00 ± 5.22 ^*#^
Diabetic + Ethyl Acetate extract (EA) 100 mg/kg	318.14 ± 5.61 ^#¥†‡^
Diabetic + Ethyl Acetate extract (EA) 200 mg/kg	312.29 ± 9.26 ^#¥†‡^
Diabetic + Dichloromethane extract (DCM) 100 mg/kg	314.57 ± 11.85 ^#¥†‡^
Diabetic + Dichloromethane extract (DCM) 200 mg/kg	325.86 ± 8.00 ^#¥†‡^
Diabetic + Hexane extract (HEX) 100 mg/kg	329.71 ± 6.51 ^#¥†‡^
Diabetic + Hexane extract (HEX) 200 mg/kg	338.86 ± 9.35 ^#¥†‡^

Data are expressed as Mean ± SEM, *n* = 7, ^#^ *p* < 0.05 versus normal control, ^*^ *p* < 0.05 compared versus diabetic control, ^¥^ *p* < 0.05 versus pioglitazone, ^†^ *p* < 0.05 versus ethanol extract 100 mg/kg, ^‡^ *p* < 0.05 versus ethanol extract 200 mg/kg.

**TABLE 2:** Effect of MA ethanol extract on insulin resistance status.

Groups	Insulin (µU/ml)	HOMA–IR	HOMA-B	TyG index	QUICKI
Normal Control (NC)	11.51 ± 0.77	2.52 ± 0.11	11.02 ± 2.86	7.99 ± 0.08	0.333 ± 0.002
Diabetic control (DC)	6.31 ± 0.48 ^#^	5.30 ± 0.27 ^#^	0.46 ± 0.06 ^#^	10.19 ± 0.04 ^#^	0.301 ± 0.002 ^#^
Diabetic + Pioglitazone (PIO) 20 mg/kg	7.06 ± 0.63 ^#^	3.17 ± 0.36 ^*^	1.20 ± 0.07 ^#^	8.93 ± 0.05 ^#*^	0.324 ± 0.005 ^*^
Diabetic + Ethanol extract (ETH) 100 mg/kg	7.70 ± 0.76 ^#^	3.65 ± 0.31 ^*^	1.20 ± 0.15 ^#^	9.13 ± 0.04 ^#*^	0.317 ± 0.004 ^#*^
Diabetic + Ethanol extract (ETH) 200 mg/kg	7.46 ± 0.90 ^#^	3.31 ± 0.31 ^*^	1.28 ± 0.21 ^#^	8.99 ± 0.03 ^#*^	0.32 1 ± 0.004 ^*^

HOMA-IR index, homeostasis model assessment for insulin resistance index; HOMA-B index, homeostasis model assessment of β-cell function; TyG index, triglyceride glucose index; QUICKI, quantitative insulin sensitivity check index. Data are expressed as Mean ± SEM, *n* = 7, ^#^ *p* < 0.05 versus normal control, ^*^ *p* < 0.05 versus diabetic control.

### Modulation of oxidative stress and antioxidant status

The cellular antioxidant defense mechanism was significantly diminished in diabetic control animals. In liver homogenate, the lipid peroxidation and oxidative stress parameter MDA was dramatically enhanced, while antioxidant indicators such as GSH, SOD, and CAT activity were significantly reduced. Administration of either Pioglitazone or MA extract significantly increased the activity of antioxidant enzymes CAT and SOD, along with GSH levels, while causing a significant decrement in MDA levels, as shown in Table 3.

**TABLE 3:** MA ethanolic extracts increase the antioxidant enzymes’ activity and decrease oxidative stress parameters.

Groups	MDA (nmol/g. tissue)	GSH (nmol/g. tissue)	CAT activity (U/g. tissue)	SOD activity (U/g. tissue)
Normal Control (NC)	259.34 ± 11.29	37.04 ± 2.31	121.03 ± 4.74	4680.14 ± 307.31
Diabetic control (DC)	429.89 ± 11.37 ^#^	14.02 ± 1.21 ^#^	55.16 ± 3.02 ^#^	2031.00 ± 193.02 ^#^
Diabetic + Pioglitazone (PIO)	339.12 ± 4.93 ^#*^	26.25 ± 0.89 ^#*^	86.65 ± 2.14 ^#*^	3502.75 ± 63.59 ^#*^
Diabetic + Ethanol extract (ETH) 100 mg/kg	327.47 ± 14.32 ^#*^	27.41 ± 2.70 ^#*^	82.73 ± 4.14 ^#*^	3649.92 ± 261.07 ^#*^
Diabetic + Ethanol extract (ETH) 200 mg/kg	305.27 ± 10.84 ^#*^	29.61 ± 2.34 ^*^	88.25 ± 7.40 ^#*^	3885.40 ± 248.02 ^*^

MDA, malondialdehyde; GSH, reduced glutathione; SOD, superoxide dismutase; CAT, catalase. Data are expressed as Mean ± SEM, *n* = 7, ^#^ *p* < 0.05 versus normal control, ^*^ *p* < 0.05 versus diabetic control.

### Effect of MA treatment on expression of T2DM related genes

**FIGURE 3:**
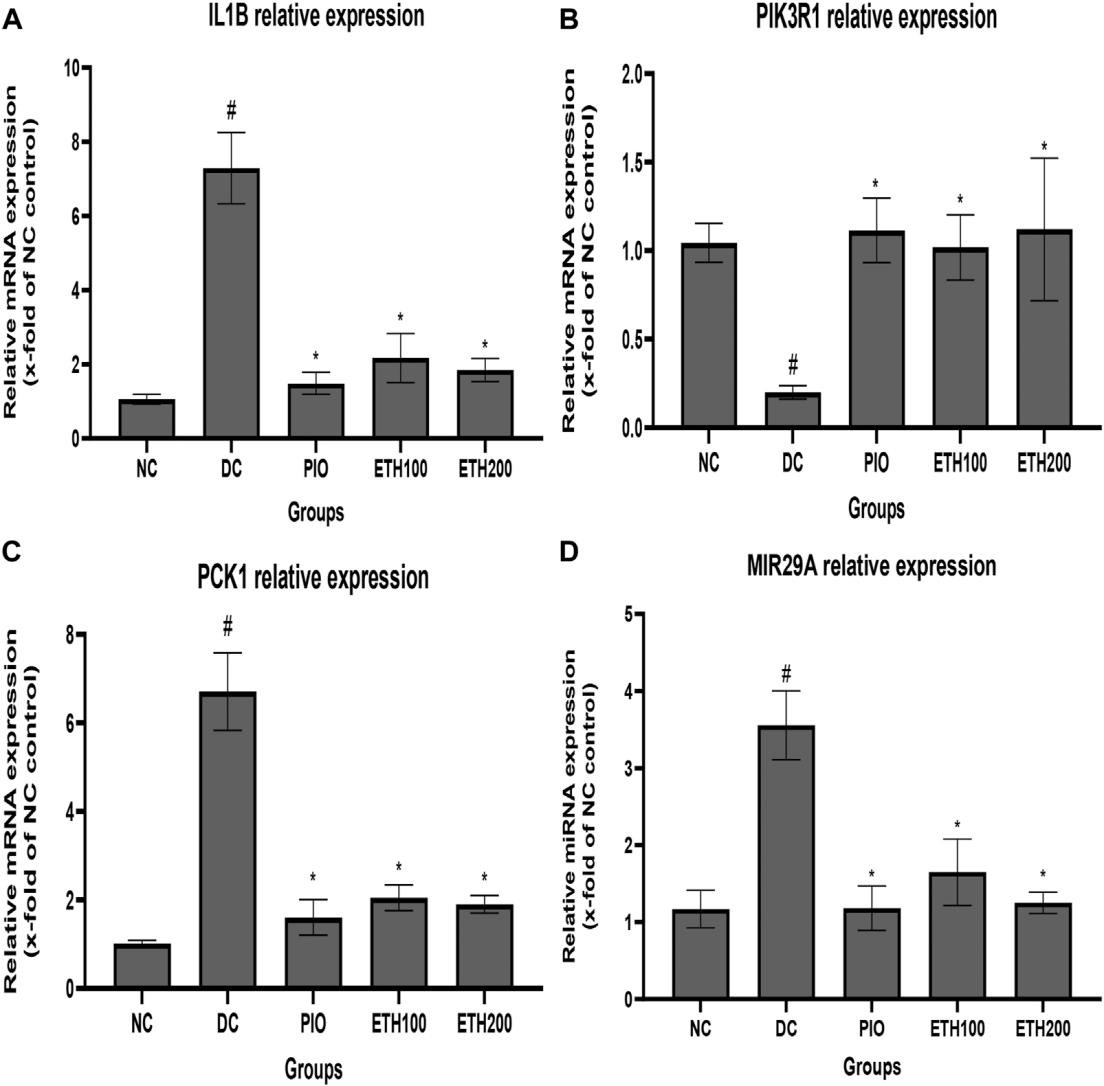
Effect of MA ethanolic extract on gene expression **(A)** MA ethanoic extract decreased the expression of the inflammatory cytokine IL1B **(B)** MA ethanoic extract increased the expression of PIK3R1. MA ethanolic extract decreased the expression of **(C)** PCK1 and **(D)** MIR -29a. Data are expressed as Mean ± SEM, *n* = 7, #*p* < 0.05 versus normal control, *p < 0.05 versus diabetic control. Adjusting the size of the images and resolution, adding the panels’ letters, and combining the images was carried out using Photoshop.

To explore the molecular mechanism of MA in the treatment of T2DM, we selected PIK3R1, PCK1, IL1B, and MIR 29A to investigate their expression level changes by qRT-PCR. As exhibited in Figure 3, the expression level of PIK3R1 was significantly downregulated, whereas the expression of PCK1, IL1B, and MIR 29A were significantly upregulated in diabetic control rats compared to normal rats. Administration of either MA extract or pioglitazone for 4 weeks was able to significantly increase PIK3R1 expression and decrease PCK1, IL1B, and MIR 29a in the treated groups compared to diabetic control rats.

### Potential bioactive compounds of MA

A total of 62 compounds were identified by utilizing UPLC/MS-MS analysis. The total ion chromatogram (TIC) is depicted in Supplementary Data Sheet S2, and the names of compounds, retention time, molecular ion and characteristic fragment masses, molecular formula, and bioavailability score are shown in Table 4. The compounds included many flavonoids and their glycosides, coumarin derivatives, stilbene derivatives, and phenolic acids. 34 compounds complied well with the drug likeness and bioavailability criteria. 28 compounds did not show good pharmacokinetic properties by violating either the rule of five or the bioavailability score or both. We ultimately retained 13 compounds out of the 28 predicted to have bad pharmacokinetic behavior because they showed important biological activity in the published literature. In addition, 15 compounds predicted to have bad pharmacokinetic behavior and one without biological targets were excluded. The final list of potential bioactive compounds in MA included 46 compounds (*See* Supplementary Table S1 for details on pharmacokinetics).

**TABLE 4:** Characterization of the chemical constituents in MA ethanolic extract using LC-ESI-MS/MS analysis.

Peak NO.	RT (min)	Adduct ion	Precursor m/z	MS/MS	Molecular formula	Compound identity	Oral bioavailability
1	1.155717	[M−H]^−^	173.0463	173, 155, 137, 111, 93, 73	C_7_H_10_O_5_	Shikimic acid	0.56
2	1.244033	[M−H]^−^	153.0212	153, 109	C_7_H_6_O_4_	Protocatechuic acid	0.56
3	1.283233	[M + H]^+^	138.0553	138, 121, 94, 77, 65	C_7_H_7_NO_2_	P-aminobenzoic acid	0.85
4	1.3062	[M−H]^−^	477.0682	477, 325, 169	C_21_H_18_O_13_	Digalloyl-shikimic acid	0.11
5	1.3312	[M−H]^−^	163.0404	119	C_9_H_8_O_3_	p-coumaric acid	0.85
6	1.343867	[M−H]^−^	289.0387	289, 259, 245, 203	C_15_H_14_O_6_	epicatechin	0.55
7	1.395033	[M−H]^−^	137.0266	137, 93, 65	C_7_H_6_O_3_	p-hydroxybenzoic acid	0.85
8	1.395033	[M−H]^−^	359.0962	359, 179	C_18_H_16_O_8_	Rosmarinic acid	0.56
9	4.12385	[M−H]^−^	177.0184	177, 149, 133	C_9_H_6_O_4_	7,8-Dihydroxycoumarin	0.55
10	5.146033	[M−H]^−^	461.1663	461, 285	C_21_H_18_O_12_	Kaempferol-3-Glucuronide	0.11
11	5.480317	[M−H]^−^	577.1572	577	C_27_H_30_O_14_	Apigenin-7-O-rhamnoglucoside (Rhoifolin)	0.17
12	5.801967	[M−H]^−^	449.0728	287, 151	C_21_H_22_O_11_	eriodictyol-7-O-glucoside	0.17
13	5.838967	[M−H]^−^	445.0768	445, 269, 175, 113	C_21_H_18_O_11_	Baicalin	0.11
14	5.897167	[M + H]^+^	449.1052	449, 431, 383, 329, 325, 299	C_21_H_20_O_11_	Luteolin-6-C-glucoside	0.17
15	5.9743	[M−H]^−^	431.0988	431, 311, 283	C_21_H_20_O_10_	Apigenin 8-C-glucoside	0.55
16	6.04665	[M + H]^+^	595.1594	595, 577, 433, 415, 337, 313, 283	C_27_H_30_O_15_	Isovitexin 7-O-glucoside (Saponarin)	0.17
17	6.16945	[M−H]^−^	408.0736	408	C_14_H_18_NO_9_S_2_^−^	Benzyl glucosinolate	0.11
18	6.5122	[M]^+^	611.1611	611, 449, 303, 299	C_27_H_31_O_16_^+^	Delphinidin-3-rutinoside	0.17
19	6.567266	[M−H]^−^	167.0357	167, 152, 108	C_8_H_8_O_4_	5-Methoxysalicylic acid	0.85
20	6.590433	[M−H]^−^	507.0946	329, 287	C_23_H_24_O_13_	Syringetin-3-O-galactoside	0.17
21	6.625433	[M−H]^−^	353.089	353	C_16_H_18_O_9_	Chlorogenic acid	0.11
22	6.6371	[M−H]^−^	623.1655	623, 315	C_28_H_32_O_16_	Isorhamnetin-3-O-rutinoside	0.17
23	6.6371	[M−H]^−^	433.0777	433, 301, 300, 271	C_20_H_18_O_11_	Quercetin-3-D-xyloside	0.17
24	6.688617	[M + H]^+^	465.1036	465, 319, 318, 147, 129, 85	C_21_H_20_O_12_	Myricitrin	0.17
25	6.828583	[M−H]^−^	447.093	447, 301, 300, 151	C_21_H_20_O_11_	Quercetin-7-O-rhamnoside	0.17
26	7.0009	[M−H]^−^	415.1244	415, 295	C_21_H_20_O_9_	Daidzein-8-C-glucoside	0.55
27	7.100234	[M−H]^−^	422.0911	422, 342, 328	C_11_H_21_NO_10_S_3_	3-(Methylsulfinyl)propylglucosinolate	0.11
28	7.1181	[M + H]^+^	193.0495	193, 178, 173, 133	C_10_H_8_O_4_	Scopoletin	0.55
29	7.274766	[M]^+^	449.1083	449, 287, 241	C_21_H_21_O_11_^+^	Cyanidin-3-glucoside	0.17
30	7.405083	[M + H]^+^	435.0908	303, 257, 229, 165, 153	C_20_H_18_O_11_	Quercetin-3-Arabinoside	0.17
31	7.51005	[M−H]^−^	317.0316	317, 179, 151, 137	C_15_H_10_O_8_	Myricetin	0.55
32	7.546717	[M−H]^−^	607.1658	607, 299, 284	C_28_H_32_O_15_	Diosmetin 7-O-rutinoside	0.17
33	7.56875	[M + H]^+^	449.1053	449, 303, 287, 257, 229, 129, 121, 71, 85	C_21_H_20_O_11_	Quercitrin	0.17
34	7.667917	[M + H]^+^	433.1139	433, 271, 153	C_21_H_20_O_10_	Apigenin-7-O-glucoside	0.55
35	7.668033	[M−H]^−^	477.1064	285	C_22_H_22_O_12_	Isorhamnetin-3-O-glucoside	0.17
36	7.668033	[M−H]^−^	431.0992	431, 317, 285, 284	C_21_H_20_O_10_	Kaempferol-3-O-alpha-L-rhamnoside	0.55
37	7.8834	[M + H]^+^	451.1967	451, 289, 225	C_21_H_22_O_11_	Okanin-4′-O-glucoside	0.17
38	7.924533	[M−H]^−^	609.188	609, 169	C_28_H_34_O_15_	Hesperetin-7-O-neohesperidoside	0.17
39	8.072383	[M + H]^+^	449.1744	449, 287, 153	C_21_H_20_O_11_	Maritimetin-6-O-glucoside	0.17
40	8.097383	[M + H]^+^	593.1944	593, 522, 473	C_28_H_32_O_14_	Acacetin-7-O-neohesperidoside	0.17
41	8.343667	[M−H]^−^	593.1942	593	C_27_H_30_O_15_	Kaempferol-7-neohesperidoside	0.17
42	8.544833	[M−H]^−^	193.0503	193, 178, 149, 134	C_10_H_10_O_4_	Ferulic acid	0.85
43	8.706984	[M−H]^−^	331.1923	331, 287	C_19_H_24_O_5_	Gibberellin A4	0.56
44	8.769983	[M−H]^−^	591.1827	591	C_28_H_32_O_14_	Acacetin-7-O-rutinoside	0.17
45	8.845984	[M−H]^−^	285.0407	175, 151, 133	C_15_H_10_O_6_	Luteolin	0.55
46	9.065467	[M−H]^−^	301.0365	301, 273, 179, 151, 121	C_15_H_10_O_7_	Quercetin	0.55
47	9.418616	[M−H]^−^	315.0527	315, 300, 271	C_16_H_12_O_7_	Isorhamnetin	0.55
48	9.6861	[M−H]^−^	271.0622	271, 177, 151, 119	C_15_H_12_O_5_	Naringenin	0.55
49	9.7896	[M−H]^−^	417.1552	417, 402, 181	C_20_H_18_O_10_	Kaempferol-3-O-alpha-L-arabinoside	0.55
50	9.946116	[M + H]^+^	169.1219	169, 151, 79, 77	C_8_H_12_N_2_O_2_	pyridoxamine	0.55
51	10.14625	[M−H]^−^	207.065	207, 178	C_11_H_12_O_4_	sinapaldehyde	0.55
52	10.17073	[M−H]^−^	269.0459	269, 225, 201, 151, 117	C_15_H_10_O_5_	Apigenin	0.55
53	10.2594	[M−H]^−^	177.0543	177, 104.7	C_9_H_6_O_4_	Esculetin	0.55
54	10.40393	[M + H]^+^	177.0546	177, 149, 133, 121, 93, 91, 77, 65	C_10_H_8_O_3_	4-methylumbelliferone	0.55
55	10.45757	[M−H]^−^	299.0547	299, 284	C_16_H_12_O_6_	Kaempferide	0.55
56	10.78905	[M−H]^−^	315.0497	315, 300	C_16_H_12_O_7_	Rhamnetin	0.55
57	11.84082	[M−H]^−^	267.0658	267, 252	C_16_H_12_O_4_	Formononetin	0.55
58	12.3858	[M−H]^−^	141.0109	141	C_2_H_6_O_3_S_2_	2-Mercaptoethanesulfonic acid	0.56
59	12.57648	[M + H]^+^	229.1435	229, 183, 119	C_14_H_12_O_3_	Resveratrol	0.55
60	13.88055	[M−H]^−^	283.0622	269, 240, 211, 151	C_16_H_12_O_5_	4′-Methoxyapigenin (Acacetin)	0.55
61	13.99208	[M + H]^+^	387.1812	121, 105	C_17_H_22_O_10_	1-O-b-D-glucopyranosyl sinapate	0.55
62	14.23207	[M + H]^+^	289.1778	289, 145, 135, 123, 117	C_15_H_12_O_6_	Eriodictyol	0.55

### Collection of therapeutic targets of MA acting on type 2 diabetes mellitus

**FIGURE 4:**
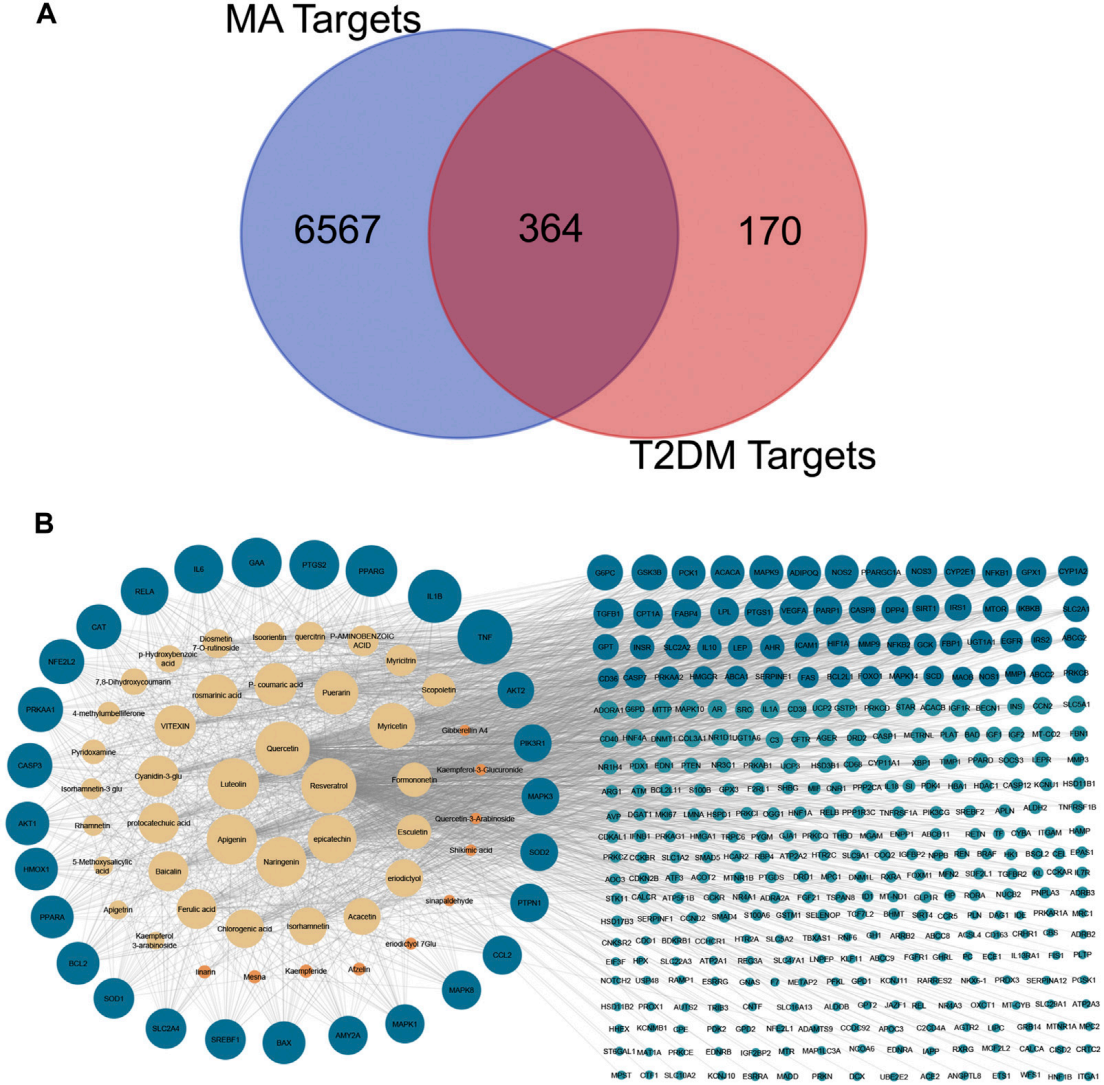
Venn diagram and compounds-targets interaction network. **(A)** Venn diagram Shows the intersection between MA targets and T2DM targets **(B)** network shows the interaction between MA phytochemicals and T2DM targets. Yellow nodes represent compounds with degrees more than the network’s 2-fold median, while orange represent compounds with degrees are less than that. Blue nodes represent targets with more than the network’s 2-fold median, while green nodes represent targets whose degrees are less than that. Adjusting the size of the images and resolution, adding the panels’ letters, and combining the images was carried out using Photoshop.

Data mining of six databases led to the collection of 6931 targets (in Supplementary Table S2), which corresponded to 46 active compounds of MA, while the total targets related to T2DM was 534 targets from four databases (in Supplementary Table S3). As shown in Figure 4A, after overlapping the herb targets with the disease targets using a Venn diagram, 364 common targets were obtained. (MA targets associated with T2DM are presented in Supplementary Table S4).

### Construction and analysis of compounds-targets network

The overlapped 364 targets were considered the potential candidate targets of MA against type 2 diabetes mellitus. 46 compounds of MA were connected to their corresponding 364 targets in a large compound-target network using cystoscope software, as shown in Figure 4B. The mathematical analysis of network topology revealed interesting information about the potential mechanisms of action. 87 therapeutic targets showed a degree ≥7 (mor than the 2-fold median degree of the network). These targets are enriched in multiple pathways related to type 2 diabetes mellitus, including adipocytokine, AMPK, TNF, HIF-1, PI3K-AKT, NOD and TOLL-like receptors, mTOR, glucagon, and insulin signaling pathways. Some of the important targets (with high degree) of MA compounds are shared with the FDA-approved medications, such as thiazolidinedione targets PPARG and PPARA, biguanides targets (AMPK) PRKAA1 and PRKAA2, alpha glucosidase inhibitors targets AMY2A and GAA, and DPP4 inhibitors targets. 37 ingredients of MA interact with ≥7 targets, suggesting a complex mechanism of MA in ameliorating type 2 diabetes mellitus and involving multi-compounds/multi-targets/multi-pathways modulation. The top 21 compounds showed a high degree ≥40 and included quercetin, apigenin, luteolin, resveratrol, epicatechin, naringenin, myricetin, *etc*. For a detailed look at compounds and target degrees, *see* Supplementary Table S5.

### Protein-protein interaction networks

**FIGURE 5:**
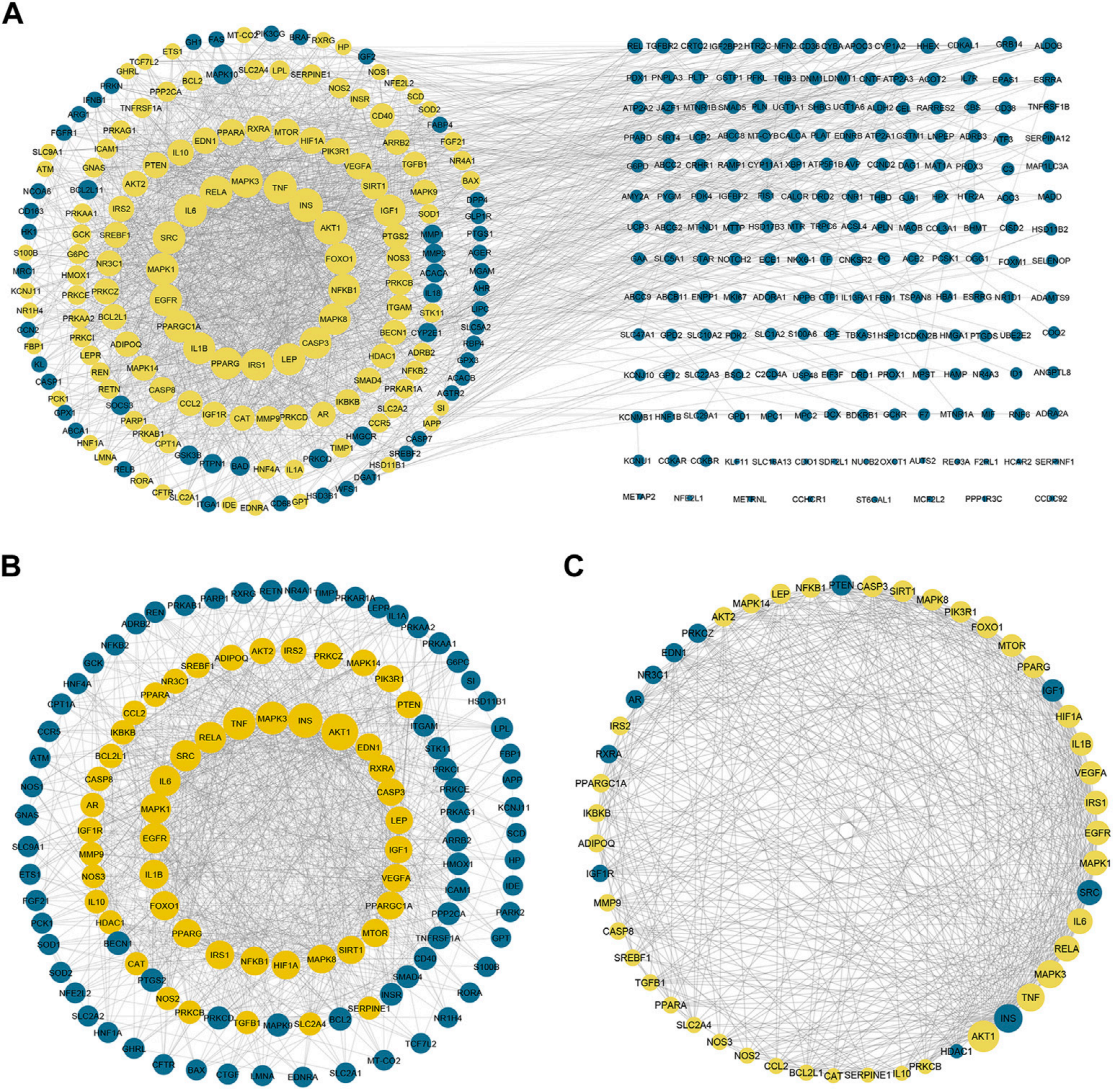
Protein-protein interaction networks. **(A)** PPI network of 363 targets of MA. Yellow nodes represent the hubs that will form the following network **(B)** represents PPI of 123 hubs whose centrality values are greater than the median. The yellow nodes represent the major hubs that will form the following network **(C)** represents PPI of the major hubs or core key targets whose centrality values are greater than the median of the hub nodes. The yellow nodes represent the core key targets that are affected by more than six compounds and will form the network of the following figure. The blue nodes in **(C)** represent targets that affected by less than seven compounds. Adjusting the size of the images and resolution, adding the panels’ letters, and combining the images was carried out using Photoshop.

**FIGURE 6:**
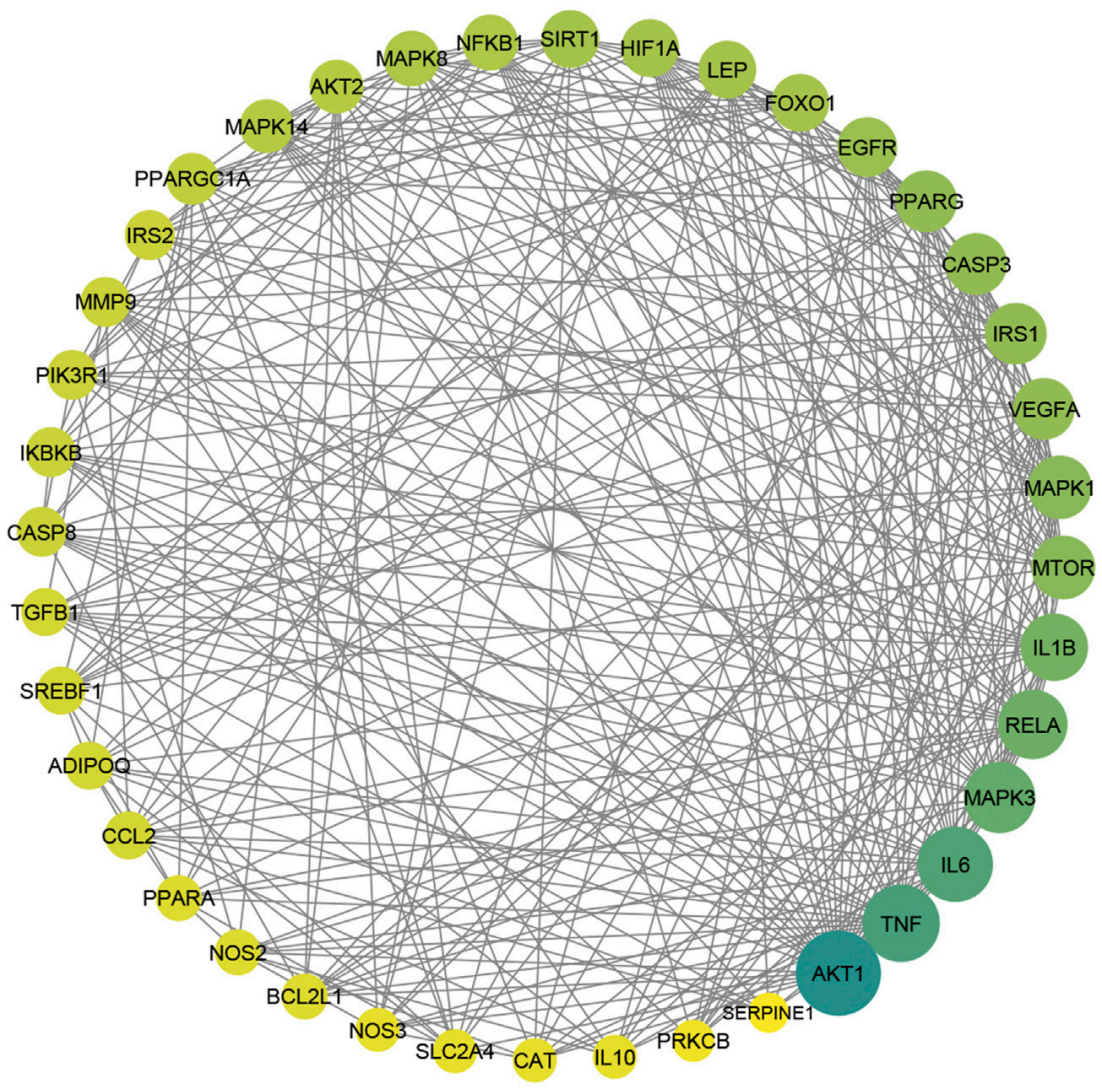
The PPI of 40 major hubs or core key targets of MA in T2DM treatment is ordered by color and size based on degree, from big and green to small and yellow, after the exclusion of the core targets affected by fewer than seven compounds. Adjusting the size of the images and resolution was carried out using Photoshop.

To gain more insight about the importance of each target and its contribution to the pharmacological mechanism of action, a protein-protein interaction network was created by importing the 364 common targets in STRING db. As shown in Figure 5A, the PPI network consisted of 363 nodes and 2856 edges (one target; CASP 12 was not identified in the String db. and was excluded). We screened the most important targets from this network in three discrete steps based on the topological properties (degree centrality, closeness centrality, and betweenness centrality) of each node. Firstly, we picked out 123 hub targets by taking nodes whose centrality values are greater than the median, as depicted in Figure 5B. Secondly, we selected 51 major hub targets whose centrality values are greater than the median value of the hubs, as shown in Figure 5C. Finally, we excluded 11 targets from the major hubs, which were affected by less than seven compounds, to end up with 40 key major hubs or core key targets for the MA mechanism in the treatment of type 2 diabetes mellitus, as shown in Figure 6. (For more details, *see* Supplementary Table S6).

### The underlying mechanism by which MA affects T2DM

**FIGURE 7:**
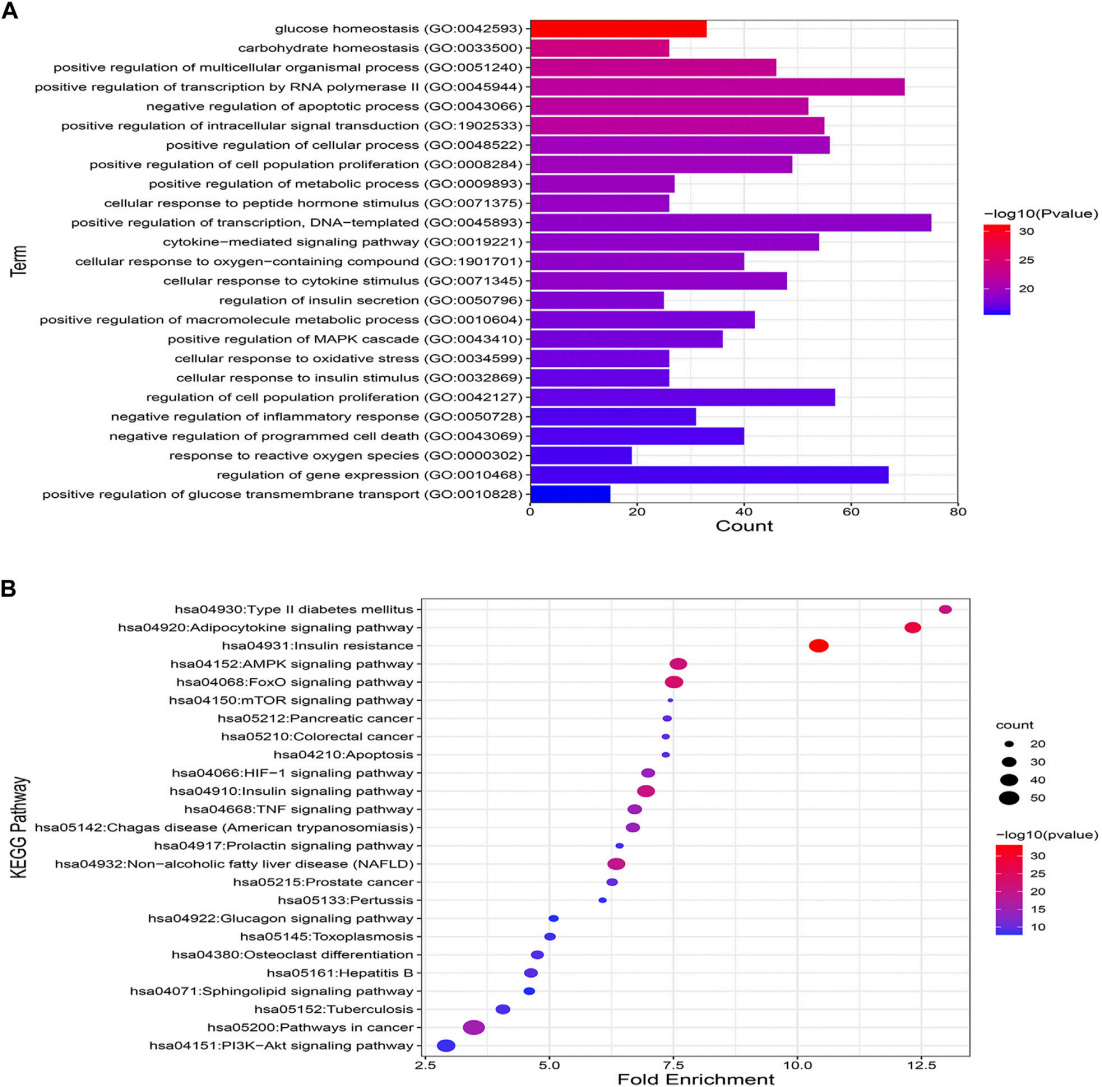
GO and KEGG pathway enrichment analyses **(A)** represents the top 25 enriched GO biological processes terms. **(B)** Represents the top 25 enriched KEGG pathways. Adjusting the size of the images and resolution, adding the panels’ letters, and combining the images was carried out using Photoshop.

Enrichment analysis based on GO and KEGG databases was performed to investigate the biological functions and pathways of MA putative target for T2DM treatment. GO analysis results suggested that MA targets are enriched in multiple biological processes linked directly to metabolism and T2DM, including glucose homeostasis, carbohydrate homeostasis, regulation of metabolic processes, cellular response to insulin stimulus, cytokine stimulus, reactive oxygen species, and oxidative stress. Figure 7A shows the top 25 enriched GO (BP) terms, and the detailed results of GO enrichment analysis, including GO (BP), GO (MF), and GO (CC), are found in Supplementary Table S7.

To further uncover the potential pathways involved in the antidiabetic effects of MA, KEGG pathway enrichment analysis was carried out. A total of 25 top enriched pathways are identified and presented in Figure 7B. More than half of the top 25 enriched pathways are well established T2DM-linked pathways. The enriched genes were linked to a variety of signaling and metabolic pathways, including energy sensing and metabolic pathways like the AMPK signaling pathway, insulin and glucagon signaling pathways, the FoXO signaling pathway, which promotes glucose homeostasis and combats oxidative stress, the HIF-1 signaling pathway, which mediates metabolic adaptation to oxygen, and immunoinflammatory pathways like the Adipocytokine and TNF signaling pathways, the PI3k-Akt signaling pathway, and pathways related to apoptosis. (For details on all KEGG enriched pathways *see* Supplementary Table S8).

**FIGURE 8:**
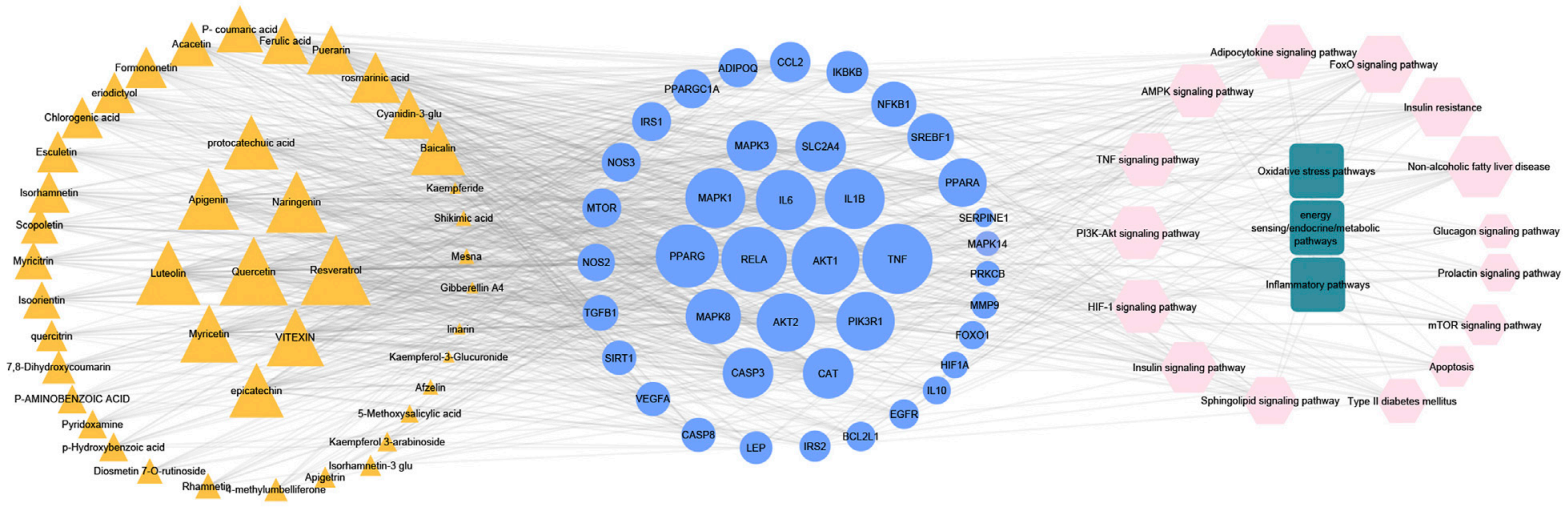
Represents compounds-core key targets-pathways network. The yellow triangles represent compounds, the blue circles represent targets, the pink Hexagons represent pathways, and the green squares represent collections of functionally related pathways. The larger the size of the node, the greater its degree. Adjusting the size of the images and resolution, adding the panels’ letters, and combining the images was carried out using Photoshop.

To get a deeper look at the enrichment analysis of all MA targets, we managed to see the relationship between the 40 core targets (major hubs) extracted from the PPI network and the key pathways related to T2DM from the enrichment analysis of all MA targets and we asked how many core targets are found in each pathway. To answer this question, we constructed a compound-core target-pathway (C-T-P) network linking MA active compounds, core targets, and pathways, as depicted in Figure 8. The analysis of the network showed that it contained 101 nodes (43 compounds, 40 core targets, and 15 pathways) and 937 edges. The importance of each pathway in this network stemmed from the number of core targets it contained, judged by degree (the number of edges between pathway node and core targets). Table 5 shows these pathways and the percentage of core targets they contain, ordered based on degree. Together, the results of computational network pharmacology and experimental work suggested that MA ameliorated T2DM through modulation of insulin resistance by multiple mechanisms, including modulation of oxidative stress pathways, inflammatory pathways, energy sensing/endocrine/metabolic pathways.

**TABLE 5:** Shows the relationship between core key targets and pathways.

No.	Pathway	Number of core targets	% Of core key targets in pathway
1	Non-alcoholic fatty liver disease	21	52.5
2	Insulin resistance	20	50
3	FoxO signaling pathway	19	47.5
4	TNF signaling pathway	17	42.5
5	Adipocytokine signaling pathway	16	40
6	HIF-1 signaling pathway	16	40
7	PI3K-Akt signaling pathway	15	37.5
8	Insulin signaling pathway	15	37.5
9	AMPK signaling pathway	14	35
10	Sphingolipid signaling pathway	13	32.5
11	Type II diabetes mellitus	11	27.5
12	Apoptosis	10	25
13	mTOR signaling pathway	10	25
14	Prolactin signaling pathway	8	20
15	Glucagon signaling pathway	7	17.5

### Molecular docking

**FIGURE 9:**
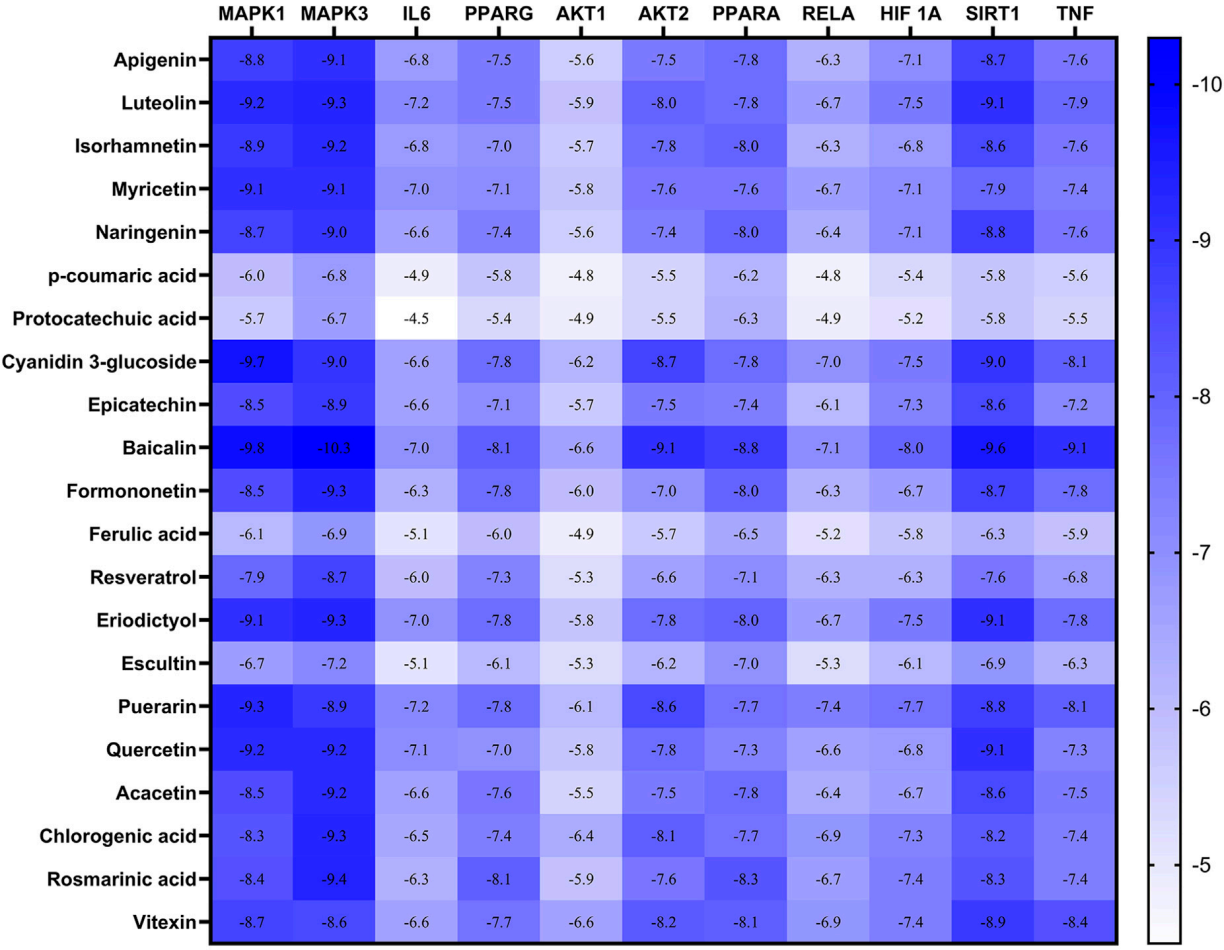
Heatmap represents the docking results and affinity of 21 compounds with 11 proteins from the core targets. The more bluish the rectangle is, the stronger the affinity. Adjusting the size of the images and resolution was carried out using Photoshop.

**FIGURE 10:**
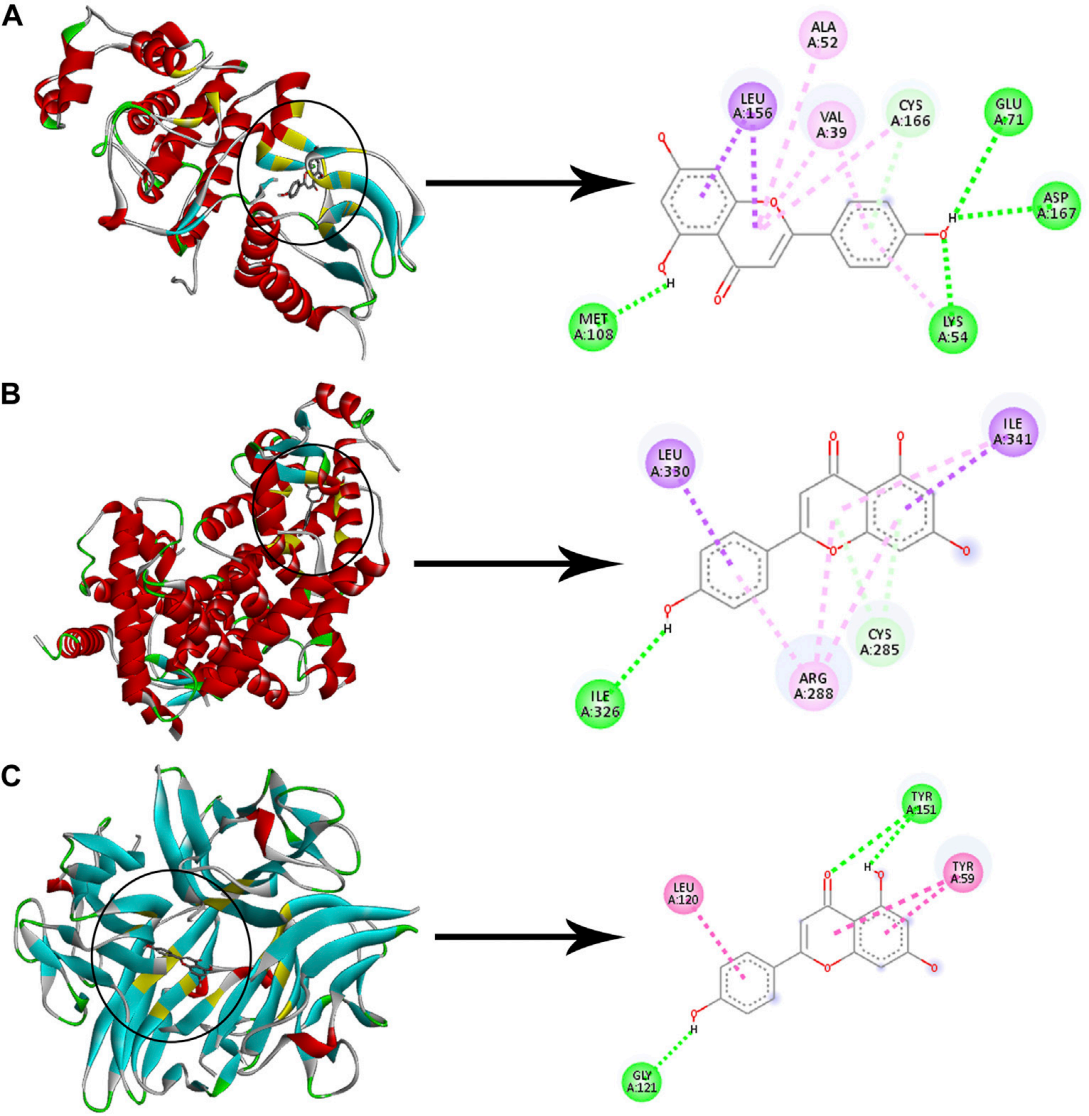
Molecular docking of apigenin with three proteins. The left images and the right images represent the 3D and the 2D structures, respectively **(A)** Docking of apigenin with MAPK1. **(B)** Docking of apigenin with PPARG. **(C)** Docking of apigenin with TNF. Adjusting the size of the images and resolution, adding the panels’ letters, and combining the images was carried out using Photoshop.

Checking the validity of network predictions against other channels of evidence, such as experimental data and computational chemistry simulation is a critical step in elucidating the mechanism of action. Molecular docking simulation is one of the most important and commonly used tools for exploring the biological activity of drugs on proteins, with affinity or binding energy being the most important parameter. The molecule with the highest biological activity has the lowest value of this parameter. The docking score values determine the binding potential or affinity of drugs to their targets, as well as the stability of conformations. A docking score value of less than −7 indicates strong binding activity, values between −5 and −7 demonstrate good binding activity, and values in the range of −4.25 to −5 signify a certain binding activity (Dong et al., 2021). Out of 40 core targets, we selected 11 proteins involved in different mechanisms to perform docking with the top 21 MA compounds (with ≥40) in terms of degree. The results are presented in Figure 9 as a heat map, and they indicate that the majority of targets predicted by network analysis have strong binding activities with the majority of tested compounds. Strong binding activity was prevalent with MAPK1, MAPK3, SIRT1, PPARA, PPARG, AKT2, TNF, and HIF1A, whereas AKT1, IL6, and RELA had good binding activity with most of the tested compounds. Generally, phenolic acids and coumarin derivatives, including p-coumaric acid, ferulic acid, protocatechuic acid, and esculetin, showed lower affinity than flavonoids. The exceptions were rosmarinic acid and chlorogenic acid, which showed a binding score pattern comparable to flavonoids. Figure 10 shows the interaction of apigenin with the active sites of MAPK1, TNF, and PPARG.

## Discussion

In humans, the progression of type 2 diabetes could be divided into two distinct stages. The first stage is the transition from a healthy metabolism to a state of declining insulin action on peripheral tissues (insulin resistance) and prediabetes. Nutritional overload and obesity are thought to be associated with the development of this state by increasing the levels of circulating triglycerides and free fatty acids that deposit in the liver, muscle, and adipose tissue, resulting in insulin resistance. Despite all the abnormalities that occur in stage 1, the pancreatic beta cells are still able to compensate for insulin resistance by producing more insulin (hyperinsulinemia). To develop frank hyperglycemia and type 2 diabetes, there must be a second stage of transition from hyperfunctional beta cells that compensate for insulin resistance to beta cell failure/death that is characterized by normal or hypoinsulinemia and the inability to compensate for insulin resistance. In animal models, a high-fat diet is typically used to cause the first stage of insulin resistance and prediabetes, while chemical inducers such as STZ are usually used to cause the second stage of pancreatic B-cell death and hypoinsulinemia. Several studies have shown that a combination of HFD and STZ for induction of T2DM in rats is a better model for mimicking the natural history of the disease (Srinivasan et al., 2005; Islam and Choi, 2007; Skovsø, 2014; Barrière et al., 2018). T2DM is a heterogeneous disorder with high morbidity and a plethora of associated abnormalities. Therefore, several proteins or pathways are linked to the development of T2DM. Antidiabetic botanical extracts contain many compounds that could induce pharmacological actions through multiple targets and signaling pathways and may help in T2DM therapy (Li et al., 2017).

*Matricaria aurea* (MA, Golden Chamomile) is a widely used herb in Middle Eastern communities for a variety of ailments, including diabetes mellitus, despite a lack of scientific evidence to support such use (Yaniv et al., 1987; Al-Mustafa and Al-Thuniba, 2008). Furthermore, a few previous studies, which were mainly *in vitro*, reported the antioxidant (Mohammad Al-Ismail and Aburjai, 2004), anti-inflammatory (Khodadadi et al., 2011), anti-ulcerative colitis (*in vivo*) (Minaiyan et al., 2011), analgesic (Qnais, 2011), antibacterial, and anticancer (Kheder et al., 2014; Ahmad et al., 2020, 2021) biological activities of *Matricaria aurea.* As far as we know, this is the first scientific study to investigate the anti-diabetic activity of MA extracts in an established type 2 diabetic rat model using HFD + STZ. The first key finding of our study is that only the polar ethanolic extract was able to reduce fasting blood glucose in diabetic rats, whereas other less polar extracts did not show any promising activity. This finding is in harmony with previous research experience that polar hydroalcoholic extraction of chamomile’s polyphenolics and flavonoids is more effective than non-polar solvents (Molnar et al., 2017; Pereira et al., 2018), and it has directed the entire research process to investigate the biological and chemical roles of MA ethanolic extract in T2DM treatment. The second key finding of our study is that MA ethanolic extract was able to significantly reduce the insulin resistance index (HOMA-IR) and the triglyceride and glucose (TyG) index. In addition, our results showed that MA ethanolic extract was not able to significantly increase the basal insulin secretion and the secretory capacity of the pancreas, as evidenced by the low level of fasting insulin and the HOMA-B index. These results indicate that MA ethanolic extract was successful in reducing insulin resistance without having a significant effect on insulin levels or the secretory capacity of the pancreas. It also suggests that MA could improve T2DM by increasing cell sensitivity to existing insulin without insulin secretagogue activity. In accordance with our findings, a previous interesting study on Roman chamomile showed that it lowers the blood glucose levels in both normal and STZ-diabetic rats without increasing the basal insulin secretion (Eddouks et al., 2005). In addition, another study examined the antihyperglycemic effect of German chamomile using STZ-diabetic rats and found that it reduces hyperglycemia without affecting insulin levels (Al-Musa and AL-Hashem, 2014). While Weidner et al. showed that German chamomile was able to reduce hyperglycemia, hyperinsulinemia, and insulin resistance in a diabetic obese model induced by long-term HFD feeding (Weidner et al., 2013). Zemestani et al. showed that chamomile can reduce insulin resistance and hyperinsulinemia in type 2 diabetic patients (Zemestani et al., 2016). Therefore, it seems that the findings of our investigation and the four previous studies support the notion that different chamomile varieties ameliorate type 2 diabetes without increasing insulin secretion or the secretory capacity of the pancreas. MA ethanolic extract was also able to significantly alleviate dyslipidemia and liver damage indicators ALT and AST, indicating an improvement in lipid metabolism and protection of the liver. The TyG index is a sensitive marker for the status of hepatic steatosis, insulin resistance, and fatty liver. Groups that received MA showed a significant improvement in their TyG index, which indicates an improvement in hepatic insulin resistance and lipid metabolism and a reduction in the lipid content of the liver (Zhang et al., 2017; Lee et al., 2019). Previous studies in HFD animal models and human clinical trials showed the ability of German chamomile to improve dyslipidemia in mice and human blood (Rafraf et al., 2015; Bayliak et al., 2021). Furthermore, it has been shown that chamomile can increase the hepatic expression of genes involved in the beta oxidation of fatty acids, including carnitine palmitoyltransferase 2, Acyl-Coenzyme A Oxidase 2, HMG-CoA synthase 2, and enoyl CoA hydratase genes. Liver damage markers in type 2 diabetes models are typically associated with steatohepatitis in liver tissue caused by a high-fat diet. The observed liver protection in MA-treated groups has also been demonstrated in several illness models, including diabetes treated with different chamomile varieties. Jabri et al. showed that chamomile can protect the liver from the lipotoxicity and oxidative stress caused by HFD in mice (Jabri et al., 2017). It has been shown that chamomile can ameliorate steatohepatitis induced by HFD, reduce the liver content of triglycerides, and prevent the rise of ALT (Mannaa et al., 2015; Bayliak et al., 2021).

Establishing the antidiabetic activity of MA ethanolic extract prompted us to search for the molecular players that might contribute to the mechanism of MA’s antidiabetic effect. Firstly, we hypothesized that the chemical components of MA ethanolic extract could influence critical T2DM targets. Using UPLC QTOF MS/MS, we identified a total of 62 chemical compounds in MA ethanolic extract. Many of the identified compounds have previously been reported to be the major flavonoids and phenolics in chamomile, including Apigenin and its glycosides, Luteolin and its glycosides, Quercetin and its glycosides, p-coumaric acid, chlorogenic acid, and ferulic acid (Aboul-Ela et al., 2012; Khan et al., 2019; Yousefbeyk et al., 2022). Then, we filtered these compounds according to their ADME properties and published pharmacological activities to obtain a final list of 46 active compounds. In this study, it was found that 46 compounds from MA could interact with 364 candidate targets related to T2DM. The analysis of the protein-protein interaction network identified 123 targets as hub proteins, including AKT1, AKT2, PIK3R1, MAPK1, RELA, PCK1, SOD1, and SOD2, *etc*. Further filtering to obtain the network’s crucial key targets revealed a total of 40 crucial core targets through which MA may exert its effect, including AKT1, AKT2, IRS1, IRS2, PIK3R1, TNF, ILB, IL6, MAPK1, MAPK3, HIF-1, and FOXO. From the rigorous interpretation of these results, some important themes started to emerge. *1*) Firstly, a single compound can regulate multiple targets on its own, whereas a single target can be regulated by many compounds; for instance, the inflammatory cytokine IL1B was modulated by more than half of all identified compounds. This observation highlights the essence of network polypharmacology and shows that simultaneously targeting numerous proteins associated with the disease may be more beneficial than targeting a single protein selectively. Moreover, the targeting of a single protein by numerous bioactive compounds suggested that MA bioactive compounds may have a synergistic effect in treating T2DM. *2*) The major constituents of MA, including apigenin, luteolin, quercetin, chlorogenic acid, p-coumaric acid, and ferulic acid, were found to target about 80% of the total targets in the network model. We think that MA’s antidiabetic activity is very likely to be mediated by the major constituents’ actions on these targets. The minor constituents might contribute to the biological activity by synergistic or additive interactions with the same targets. Experimental studies of synergy are needed to validate or rule out this assumption. *3*) Analysis of the relationships between the crucial core targets predicted by network analysis and the enriched pathways revealed that each of the top enriched pathways contains a large number of the core targets; for example, more than one-third of the core targets are found in the insulin signaling pathway and approximately half of the core targets are found in the Foxo signaling pathway. To emphasize this point, we think that the aggregation of a large proportion of the core targets predicted by PPI network analysis in the top pathways reflects the powerful prediction capability and reliability of such networks for inferring action mechanisms. *4*) The fourth pattern revealed by network analysis was the ability of one target to be involved in more than one pathway, implying crosstalk and a close relationship between these pathways. For example, PIK3R1 is involved in nine of the most important pathways related to T2DM, such as the insulin signaling pathway, the FoxO signaling pathway, and so on. This observation captured network biology’s realization of the modularity nature of complex diseases such as diabetes. The importance of targets in this model stems from their importance in the disease module, whereas the importance of drugs stems from their ability to modulate essential hubs in the disease module. Moreover, GO and KEGG pathway enrichment analyses of the putative targets identified multiple pathways, mainly inflammatory pathways, oxidative stress pathways, and endocrine/energy-sensing/metabolic pathways.

Inflammation is widely recognized as a crucial factor in the pathogenesis of T2DM and insulin resistance. Local inflammation in the peripheral tissues plays a significant role in the vicious cycle of diabetes abnormalities (Cai et al., 2005; Catrysse and van Loo, 2017). Reducing inflammation by blocking or decreasing the effect of the inflammatory mediator IL-1B has drawn the attention of many researchers interested in discovering newer antidiabetics. Treatment with IL1 receptor antagonists has been shown to decrease hyperglycemia, enhance B cell function and increase insulin sensitivity in peripheral tissues, notably the liver (Ehses et al., 2009). Our network analysis predicted that the inflammatory cytokines IL1B, IL6, and TNF are crucial targets for MA in treating T2DM. This prediction is consistent with the mRNA expression of IL1B that was downregulated in the MA treated groups compared with the diabetic non-treated animals. In agreement with our experimental results and network predictions, a previous study showed that chamomile was able to mitigate inflammation and reduce the expression of inflammatory genes in the liver of insulin-resistant obese mice (Weidner et al., 2013). In addition, previous studies showed that chamomile reduces the inflammatory markers in the blood of animal models and human type 2 diabetes patients (Shelbaya, 2017; Nargesi et al., 2018; Zemestani et al., 2018). Furthermore, several studies showed the effectiveness of chamomile in mitigating inflammation in various inflammatory disease models, including macrophages, brain, skin, colon, and adipocytes. (Srivastava et al., 2009; Bhaskaran et al., 2010; Curra et al., 2013; Menghini et al., 2016; Ortiz et al., 2017; Russo et al., 2021; Wang et al., 2021; Jabri et al., 2022). A deeper explanation for the observed anti-inflammatory effect of MA emerged from the appearance of RELA (NFKB 65), NFKB1, MAPK1, and MAPK3 as core targets in the network. Obesity, nutritional overload, and oxidative stress associated with T2DM have been shown to activate these signaling proteins, leading to the development of inflammation and insulin resistance in peripheral tissues (Catrysse and van Loo, 2017). Inhibition of RELA, NFKB1, MAPK1, and MAPK3 may mitigate inflammation and reduce inflammatory cytokines expression. In accord with our model predictions, previous studies showed that major chamomile flavonoids, including apigenin, luteolin, and quercetin, have powerful anti-inflammatory activity through inhibiting the activation of RELA (NFKB65) and MAPK proteins and reducing their expression (Malik et al., 2017; Choy et al., 2019). Furthermore, ferulic acid and chlorogenic acid have been shown to reduce proinflammatory mediators by inhibiting the MAPK and NFKB pathways (Bao et al., 2018; Ghosh et al., 2018; Chowdhury et al., 2019; Yan et al., 2020). In addition, the anti-inflammatory activity and reduction of IL1B and TNF expression are known with p-coumaric acid treatment (Alam et al., 2016; Mozaffari Godarzi et al., 2020). Molecular docking simulation gave a booster support for this mechanism, showing a strong binding energy with MAPK1, MAPK3 (less than −8.5 with most compounds), and TNF, and a good binding energy with IL6 and RELA. Furthermore, the study of Bhaskaran et al. on the effect of chamomile on inflammatory mechanisms in macrophages lent great support to our predicted mechanism through RELA/NFKB65. This study demonstrated that chamomile was able to reduce LPS-induced NO production and block IL1B, TNF, and IL6-induced NO formation in macrophages through inhibition of RELA/NFKB65 activation and iNOS gene expression (Bhaskaran et al., 2010). MMP9 is another inflammatory protein predicted to be a major hub modulated by MA in the PPI network. This prediction is consistent with an *in vitro* study that showed the anti-inflammatory activity of MA hydroethanolic extract *via* inhibiting MMPs proteins (Khodadi et al., 2011). Bulgari and others showed that the anti-inflammatory effect of chamomile at the gastric level was mediated through inhibition of metalloproteinase 9 (MMP9) and the NFKB pathway (Bulgari et al., 2012). According to the results of GO and KEGG pathway enrichment analyses, the pathways related to inflammation, including TNF, Adipocytokine, Toll-like receptor, and MAPK signaling pathways, are major pathways through which MA compounds can modulate the inflammatory processes. The combined experimental and computational evidence suggests that an anti-inflammatory mechanism is involved in ameliorating T2DM by MA ethanolic extract.

Oxidative stress is one of the major factors in the pathogenesis of T2DM. Flavonoids and polyphenols’ actions are frequently attributed to their antioxidative capacity. The outcomes of our study showed a significant improvement in the antioxidant enzymes CAT and SOD activity, as well as GSH content in the liver. In agreement with our results, many studies have shown the antioxidative stress potential of different Matricaria species in either *in vitro* or *in vivo* models. It has been shown that chamomile diminishes the lipotoxicity and oxidative stress markers in the liver of HFD-fed rats (Mohammad Al-Ismail and Aburjai, 2004; Cemek et al., 2008; Jabri et al., 2017; Al-Dabbagh et al., 2019). Moreover, chamomile tea was successful in reducing oxidative stress markers in the blood of diabetic human subjects (Zemestani et al., 2016). Network analysis provided a deeper underlying mechanism for increasing the antioxidant potential through the induction of Nrf2 (gene name: NFE2L2). Nrf2 is a transcription factor that induces antioxidant genes *via* interaction with the antioxidant response element (ARE) in DNA, and hence plays a central role in the defense system of the cell in combating oxidative stress. Nrf2 regulates the transcription of antioxidative stress enzymes such as CAT, SOD, HMOX1, and glutathione peroxidase (David et al., 2017). More than 20 of the identified compounds were shown to modulate Nrf2. Another probable mechanism involves modulation of FoxO signaling and SIRT1. In accordance with our results, previous studies showed the ability of various flavonoids, including Apigenin, Luteolin, quercetin, naringenin, and myricetin, to act as modulators of Nrf2 and FoxO (Pallauf et al., 2017). Furthermore, hydroxycinnamic acid derivatives such as chlorogenic acid and ferulic acid have been shown to protect against hyperglycemia-induced oxidative stress *via* activation of Nrf2 (Song et al., 2016; Bao et al., 2018). On the other hand, Baicalin has been demonstrated to upregulate SOD and CAT through modulation of FoxO phosphorylation (Kim et al., 2012). In the case of Formononetin, it has been shown to indirectly upregulate Nrf2 *via* SIRT1 (Zhuang et al., 2021). In accord with our model predictions, a couple of studies have shown that chamomile can protect macrophages from oxidative stress through induction of Nrf2 accumulation, leading to an increase in antioxidant enzymes levels (Bhaskaran et al., 2012, 2013). The correlation between the experimental antioxidative potential of MA extract and network prediction of important proteins that regulate oxidative stress refers to an antioxidant mechanism involved in the amelioration of T2DM.

T2DM is associated with insulin resistance, defective insulin signaling, and unrestrained gluconeogenesis. PCK1 is the rate-limiting enzyme in gluconeogenesis, which is transcriptionally controlled by insulin. In the diabetic state, the normal insulin signal that goes through INSR, IRS1, IRS2, PI3K, AKT, and Foxo to inhibit PCK1 transcription and reduce gluconeogenesis is aborted (Peng et al., 2020; Ge et al., 2021). Our study showed that the insulin signaling pathway was modulated by MA administration. Our network analysis indicated that the majority of this pathway’s components are either hubs, such as INSR and PCK1, or core targets, such as IRS1, IRS2, PIK3R1, AKT1, AKT2, and Foxo. Treatment with MA led to significant upregulation of PIK3R1 mRNA expression and significant downregulation of PCK1 expression compared to diabetic control, which signifies improvement in insulin signaling and inhibition of gluconeogenesis. In agreement with our results, a previous mechanistic study found that popular chamomile flavonoids Apigenin and luteolin were able to inhibit gluconeogenesis and PCK1 expression *via* modulation of FOXO1, AKT, and Nrf2 (Bumke-Vogt et al., 2014), while Liu et al. demonstrated that Daidzein-8-C-glucoside (Puerarin) could upregulate PI3K, AKT, pAKT and pfoxo1, leading to downregulation of PCK1 and inhibition of gluconeogenesis (Liu et al., 2021). Ferulic acid has been shown to attenuate glucose production by inhibiting PCK1, G6Pase, and glycogen phosphorylase and increasing the activity of glucokinase and glycogen synthase, while chlorogenic acid inhibits glucose 6-phosphatase and gluconeogenesis (Alam et al., 2016). The effect of chlorogenic acid was mediated through modulation of IRS1, PI3K, AKT, GSK3β, and FOXO1 signaling proteins (Gao et al., 2018; Yan et al., 2020). Other previous studies have shown that luteolin, quercetin, and apigenin could improve insulin signaling *via* modulation of PI3K (Nepali et al., 2015; Kalivarathan et al., 2020; Eraky et al., 2022). The second important mechanism predicted by network analysis was the activation of the AMPK signaling pathway, which contributes to explaining the observed modulation of glucose and lipid metabolism. Insulin-independent activation of AMPK suppresses the transcription factors Foxo1 and CRTC2, which are responsible for PCK1 transcription and gluconeogenesis. Furthermore, AMPK signaling inhibits gluconeogenesis by decreasing PPAR coactivator 1α (gene name: PPARGC1A) expression and increasing phosphorylation of glycogen synthase kinase 3 (GSK-3) (Joshi et al., 2019). Moreover, AMPK increases fatty acid oxidation in the liver and decreases *de novo* fatty acid synthesis, cholesterol synthesis, and lipogenesis by inhibiting sterol regulatory element binding protein 1c (SREBF1), HMG CoA reductase (HMGCR), and Acetyl-coA carboxylase (ACACA). In agreement with our proposal, previous studies have shown that MA ingredients, including Apigenin, luteolin, quercetin, ferulic acid, chlorogenic acid, isoorientin, naringenin, and resveratrol, ameliorate hyperglycemia and hyperlipidemia through activation of the AMPK signaling pathway and SIRT1 (Ong et al., 2012; Jin et al., 2015; Tsai et al., 2018; Joshi et al., 2019; DiNicolantonio et al., 2022). Our network analysis also revealed an increase in adiponectin and leptin production, which may activate AMPK signaling. In agreement with our predictions, previous studies have shown that apigenin, quercetin, and chlorogenic acid increase the adiponectin and leptin levels (Nisha et al., 2014; Jin et al., 2015; Zhang et al., 2018). Our study added a new layer of complexity to the process of improving the insulin signaling pathway through modulation of epigenetic mechanisms. Mir29A has been reported to be a critical regulatory hub in T2DM and its upregulation inhibits the insulin signal by targeting PIK3R1 and IRS1, resulting in uncontrolled gluconeogenesis and PCK1 upregulation (Pandey et al., 2011; Kurtz et al., 2014; Yang et al., 2014; Dooley et al., 2016). The q-RTPCR expression analysis revealed that both pioglitazone and MA extracts were effective in normalizing MIR29A expression in the liver, suggesting another epigenetic modulation of insulin resistance by MA. A recent study identified morin, a bioflavonoid closely related to MA flavonoids, as an inhibitor of MIR29A upregulation in human HepG2 cells (Razavi et al., 2019). The experimental and computational data suggest that MA ethanolic extract ameliorates T2DM by modulating multiple metabolic pathways, including insulin, FoxO, AMPK, and PI3K-AKT signaling pathways.

Network pharmacology analysis showed that some popular metabolic targets for the FDA-approved antidiabetic drugs, such as PPARG and PPARA, the targets of pioglitazone, might play a great role in MA activity against insulin resistance and dyslipidemia associated with T2DM. In the cases of PPARG and PPARA, the molecular docking scores referred to a strong binding with the majority of tested compounds (less than −7). In perfect agreement with our model’s predictions, a mechanistic study showed that chamomile ameliorates insulin resistance through transcriptional stimulation of PPARG and PPARA in the adipose tissue and liver, respectively (Weidner et al., 2013). In addition, previous research has shown that flavonoids can modulate PPARG and PPARA activity, which is consistent with our prediction. According to Feng et al., apigenin improves insulin resistance in muscle and liver and attenuates metabolic inflammation through PPARG activation (Feng et al., 2016), while Beekman et al. indicated that quercetin and kaempferol increase PPARG receptor mRNA expression. Other studies using apigenin, luteolin, chlorogenic acid, naringenin, formononetin, myricetin, diosmetin, and baicalin found a similar activation of PPARG-mediated gene expression (Beekmann et al., 2015; Sanchez et al., 2017; Peng et al., 2018). Moreover, previous studies showed that apigenin, quercetin, chlorogenic acid, rosmarinic acid, naringenin, Hesperitin, daidzein, formononetin, and umbelliferone can activate and/or increase the expression of PPARA (Shi et al., 2015; Alam et al., 2016; Rigano et al., 2017; Sanchez et al., 2017; Wang et al., 2019; Doi et al., 2022). Network analysis showed that alpha glucosidase and alpha amylase enzymes are potential targets for a large number of MA compounds. Prior investigation showed the ability of apigenin, quercetin, luteolin, chlorogenic acid, ferulic acid, and protocatechuic acid to inhibit alpha glucosidase and alpha amylase in *in vitro* studies (Malunga et al., 2018; Takahama and Hirota, 2018; Şöhretoğlu and Sari, 2020; Aleixandre et al., 2022). In perfect agreement with our predictions, previous investigations showed that chamomile can reduce carbohydrate digestion and absorption by inhibiting alpha glucosidase and alpha amylase enzymes (Kato et al., 2008; Villa-Rodriguez et al., 2018).

It is very interesting to find that natural compounds can modulate the targets of FDA-approved drugs, although they are chemically different from each other. According to Liu and others, the calculated chemical similarity scores between FDA-drugs and popular flavonoids were less than 0.6 for the majority of FDA-approved antidiabetic drugs, except some SGLT2 inhibitors (liu et al., 2020). We conducted a similarity score calculation using RDKIT (unpublished data) and found that it was less than 0.6.

Hypoxia and impaired adaptive responses to hypoxia in diabetic tissues as a result of inadequate HIF-1 stimulation seem to be pathogenic agents in the development of diabetes and its afflictions. However, the detailed mechanism has not yet been discovered (Catrina and Zheng, 2021). The outcomes of network analysis showed that HIF-1 is a major hub in the PPI network and might contribute to the antidiabetic mechanisms of MA. The molecular docking scores of MA compounds with HIF-1 were very good and suggested a probable activity. A previous study indicated that quercetin activates and increases the accumulation of HIF-1α in normoxia. However, other studies in cancer models showed the opposite effect with apigenin and quercetin, suggesting a complex role of HIF-1 in different health states (Wilson and Poellinger, 2002; Park et al., 2008; Samec et al., 2021). More studies are needed to detect the exact role and molecular mechanism of HIF-1 in the diabetic state and how it could be exploited as a therapeutic target.

The limitation of our study lies in its inability to dictate the extent to which every single mechanism contributes to the final observed phenotype. It is possible that the proposed different mechanisms collectively contribute and cooperate to improve the disease state. However, it is very likely that some regulated mechanisms contribute more to treatment than their rivals. These issues can be resolved in the future by further detailed mechanistic studies. Another limitation is the small number of animals used in the experiment. However, if we consider the large effect size needed to be detected (in blood glucose and other parameters), we will find that the small sample size is sufficient. Before considering this plant for advanced clinical trials in humans, larger studies using a higher number of animals are warranted in future studies. In addition, the network model in our study was based on the chemical profile of MA ethanolic extract, taking into account the minor constituents’ interactions. The role of minor constituents needs to be validated or refuted in further synergy studies.

## Conclusion

In this study, we established for the first time the antidiabetic potential of *Matricaria aurea.* Integrating different channels of evidence: experimental, computational biology, and computational chemistry, helped us to elucidate the general action mechanisms that mediate MA antidiabetic activity at the molecular level from a systems biology perspective. This mechanism can be summarized in modulation of inflammatory pathways, energy sensing/endocrine/metabolic pathways, as well as oxidative stress pathways. MA can serve as a source of new antidiabetics and nutraceutical formulations to improve diabetic state.

## Data Availability

The original contributions presented in the study are included in the article/Supplementary Material, further inquiries can be directed to the corresponding author.
