# Supplementary material for: Integrating experimental model, LC-MS/MS chemical analysis, and systems biology approach to investigate the possible antidiabetic effect and mechanisms of Matricaria aurea (Golden Chamomile) in type 2 diabetes mellitus
Source: Front Pharmacol. 2022 Sep 7;13:924478. doi: 10.3389/fphar.2022.924478 (PMC9490514; doi:10.3389/fphar.2022.924478)
Supplement: Supplementary file 1 [file DataSheet2.PDF]

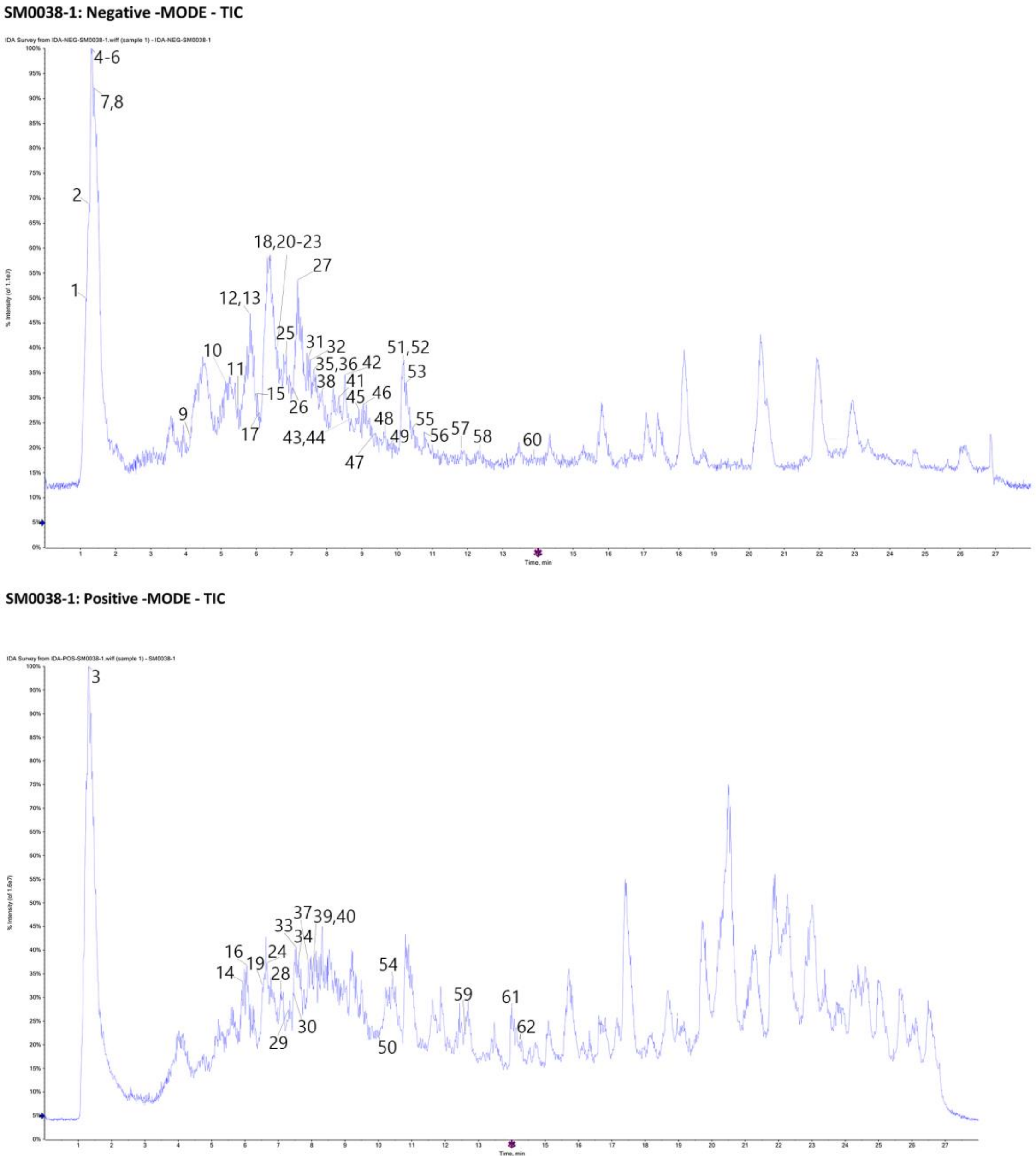

Figure 1. The total ion chromatogram of MA ethanolic extract. **(A)** the total ion chromatogram in negative mode **(B)** the total ion chromatogram in positive mode. Adjusting the size of the image, resolution, and adding numbers and lines to match the compounds in the table with the peaks of the total ion chromatogram was carried out using Photoshop.
