# Supplementary material for: Integrating experimental model, LC-MS/MS chemical analysis, and systems biology approach to investigate the possible antidiabetic effect and mechanisms of Matricaria aurea (Golden Chamomile) in type 2 diabetes mellitus
Source: Front Pharmacol. 2022 Sep 7;13:924478. doi: 10.3389/fphar.2022.924478 (PMC9490514; doi:10.3389/fphar.2022.924478)
Supplement: Supplementary file 5 [file DataSheet1.PDF]

Table 1. Composition and energy density of normal diet and high-fat diet.

Ingredients	Normal diet (g)	High-fat diet (g)
Cornstarch	54.32	15.00
Sucrose	10	7.5
Casein	20.16	33
Corn oil	5.12	3.1
Lard	0	31
Cellulose	5	5
Vitamin mix	1	1
Mineral mix	4	4
L- Methionine	0.3	0.3
Salt	0.1	0.1
Total	100 g	100 g
Energy density, Kcal/100gm	384	529
